# Large area imaging of forensic evidence with MA-XRF

**DOI:** 10.1038/s41598-017-15468-5

**Published:** 2017-11-08

**Authors:** Kirsten Langstraat, Alwin Knijnenberg, Gerda Edelman, Linda van de Merwe, Annelies van Loon, Joris Dik, Arian van Asten

**Affiliations:** 1 Netherlands Forensic Institute, P.O. Box 24044, 2490 AA The Hague, The Netherlands; 2 Rijksmuseum, P.O. Box 74888, 1070 DN Amsterdam, The Netherlands; 3 Delft University of Technology, Materials Science and Engineering, P.O. Box 5, 2600 AA Delft, The Netherlands; 4 University of Amsterdam, Faculty of Science, Van’t Hoff Institute for Molecular Sciences, P.O. Box 94157, 1090 GD Amsterdam, The Netherlands; 5 CLHC, Amsterdam Center for Forensic Science and Medicine, P.O. Box 94157, 1090 GD Amsterdam, The Netherlands

## Abstract

This study introduces the use of macroscopic X-ray fluorescence (MA-XRF) for the detection, classification and imaging of forensic traces over large object areas such as entire pieces of clothing and wall paneling. MA-XRF was sufficiently sensitive and selective to detect human biological traces like blood, semen, saliva, sweat and urine on fabric on the basis of Fe, Zn, K, Cl and Ca elemental signatures. With MA-XRF a new chemical contrast is introduced for human stain detection and this can provide a valuable alternative when the evidence item is challenging for conventional techniques. MA-XRF was also successfully employed for the chemical imaging and classification of gunshot residues (GSR). The full and non-invasive elemental mapping (Pb, Ba, Sr, K and Cl) of intact pieces of clothing allows for a detailed shooting incident reconstruction linking firearms and ammunition to point of impact and providing information on the shooting angle. In high resolution mode MA-XRF can even be used to provide information on the shooting order of different ammunition types. Finally, by using the surface penetration of X-rays we demonstrate that the lead signature of a bullet impact can be easily detected even if covered by multiple layers of wall paint or human blood.

## Introduction

The introduction and use of macroscopic (or macro) X-ray Fluorescence (MA-XRF) imaging has shown great value in the study of art objects^1–3^. Recent instrumental developments enable MA-XRF analysis directly at the museum using mobile XRF scanners and allow for the elemental mapping of large surfaces. The experiences with a commercially available MA-XRF instrument also have shown potential in terms of repeatability and reproducibility^1^. Because X-rays penetrate the surface to some extent, depth profiling is also often feasible enabling art historians, conservators and scientists to visualize and study hidden layers beneath the surface of well-known paintings and other art objects. This invaluable source of concealed information with respect to the artist’s working methods, art authentication and conservation is retrieved in a fully non-invasive manner and in a single scan^4^. XRF scanning has been demonstrated and successfully applied in forensic science and archeology but until now only for moderate surface areas with 2D micro XRF (2D μ-XRF). This limits the elemental mapping to a localized area for traces such as fingerprints^5^, microparticles^6^, hair^7^ and metal residues^8^. However, the majority of the forensic studies involving μ-XRF scanning has focused on gun shot residues^9–16^. This is understandable given the fact that the typical heavier GSR elements can be detected with high sensitivity and selectivity with XRF. To our knowledge this study is the first to demonstrate the forensic potential of MA-XRF (*i.e*. large area XRF imaging) through a number of proof of principle experiments. Elemental imaging was applied to entire pieces of clothing and wall paneling to detect, visualize, characterize and classify human biological traces and gunshot residues. MA-XRF provides an interesting additional ‘elemental contrast’ to imaging when conventional optical and fluorescence methods involving forensic light sources and IR/NIR based hyperspectral imaging are hampered by substrate interference (*e.g*. limited reflection on dark clothing^17–19^ or excessive background fluorescence^20^). As alternative for the more invasive chemographic methods^21^ for shooting distance estimation (important when investigating opposing scenarios, *e.g*. wrestling leading to an accidental gunshot versus a close-range execution) MA-XRF potentially provides valuable additional information for complex shooting incident reconstruction involving multiple firearms, ammunition types and shooters. This could even be the case in situations where perpetrators have attempted to conceal evidence at the scene in the aftermath of a crime. In a fashion similar to the analysis of hidden features in works of art, the potential of MA-XRF was investigated to detect bullet lead residues covered by human blood and multiple layers of blue wall paint. In situations where police officers have indications of crime concealment or covered traces, the use of MA-XRF as a mobile tool directly at the scene of crime could therefore be an interesting future prospect.

## Results

### Detection of human biological traces on clothing through elemental mapping

The ability of MA-XRF imaging to detect biological traces of forensic relevance is mostly governed by its limited sensitivity in relation to relatively low levels of marker elements (see Supplementary Information, Table S1 for an overview of typical levels of the main elements in human biological matrices).

If a biological perpetrator stain originating from 1 drop of blood, semen, saliva, sweat or urine is considered with a volume of 50 µl this corresponds to an amount of 5–200 µg of an element of XRF interest. If this drop is deposited on a piece of clothing of the victim or at the crime scene and results in a stain with a surface area of approximately 1 cm^2^ (this will be affected by fluid, substrate and environmental characteristics), this will yield average elemental surface concentrations of 5–200 µg/cm^2^. Ideally, MA-XRF detection limits for the elements of interest should be a factor of 10 lower (0.5–20 µg/cm^2^) to be able to confidently image the marker elements.

To determine whether MA-XRF sensitivity would be sufficient for the successful detection and imaging of crime related human biological stains of realistic forensic dimensions and mass, calibration curves were constructed. This was done for three elements, *i.e*. Fe, Zn and Pb (an element mainly of interest for GSR imaging as discussed in the next paragraph) on the M4 XRF scanner for conditions as similar as possible to the large area M6 XRF setup. Good linearity was obtained for all elements in the range of 10–10000 µg/ml (See Supplementary Information, Figure S1) with detection limits of 1.5 µg/ml for Fe, 2.2 µg/ml for Zn and 15.3 µg/ml for Pb. With the methodology described in the sample preparations section this corresponds to surface concentrations limits of, respectively, 0.2 µg/cm^2^, 0.3 µg/cm^2^ and 2 µg/cm^2^. This indicates that the current MA-XRF instruments are sensitive enough to effectively detect biological traces through marker elements such as Fe and Zn. These results were obtained for 1800 measurement pixels covering the entire calibration spot of 5.3 cm^2^. When reducing the XRF scan area the signal intensity remains constant but the noise levels start to increase leading to an increase in the detection limit with a factor of 6 for an area covering 25 measurement pixels (corresponding to roughly 0.06 cm^2^). This phenomenon (illustrated in Figure S2 of the Supplementary Information) seems to be governed by elementary noise statistics and indicates that a trade-off exists between scan area and elemental concentrations. Smaller stains can only effectively be detected and imaged when the monitored element is present at relatively high (surface) concentrations.

**Figure 1:**
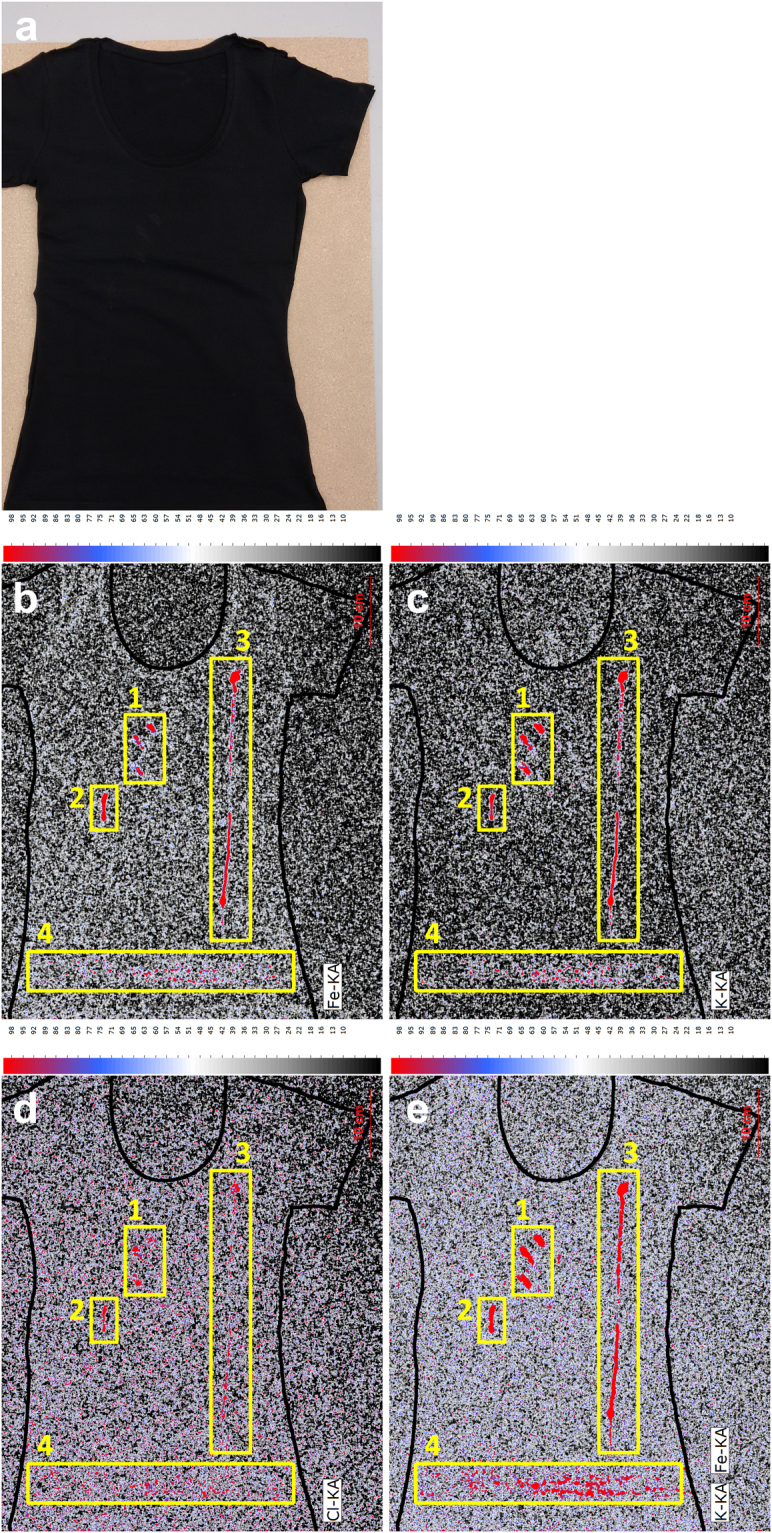
MA XRF elemental scans of Fe (**b**), K (**c**) and Cl (**d**) and a combined element map (**e**) of a black T-shirt (**a**) containing several blood patterns (*i.e*. (1) transfer pattern, (2) bloody nose, (3) expirated blood and (4) impact pattern) applied under controlled conditions (pixel scan time = 35 ms).

**Figure 2:**
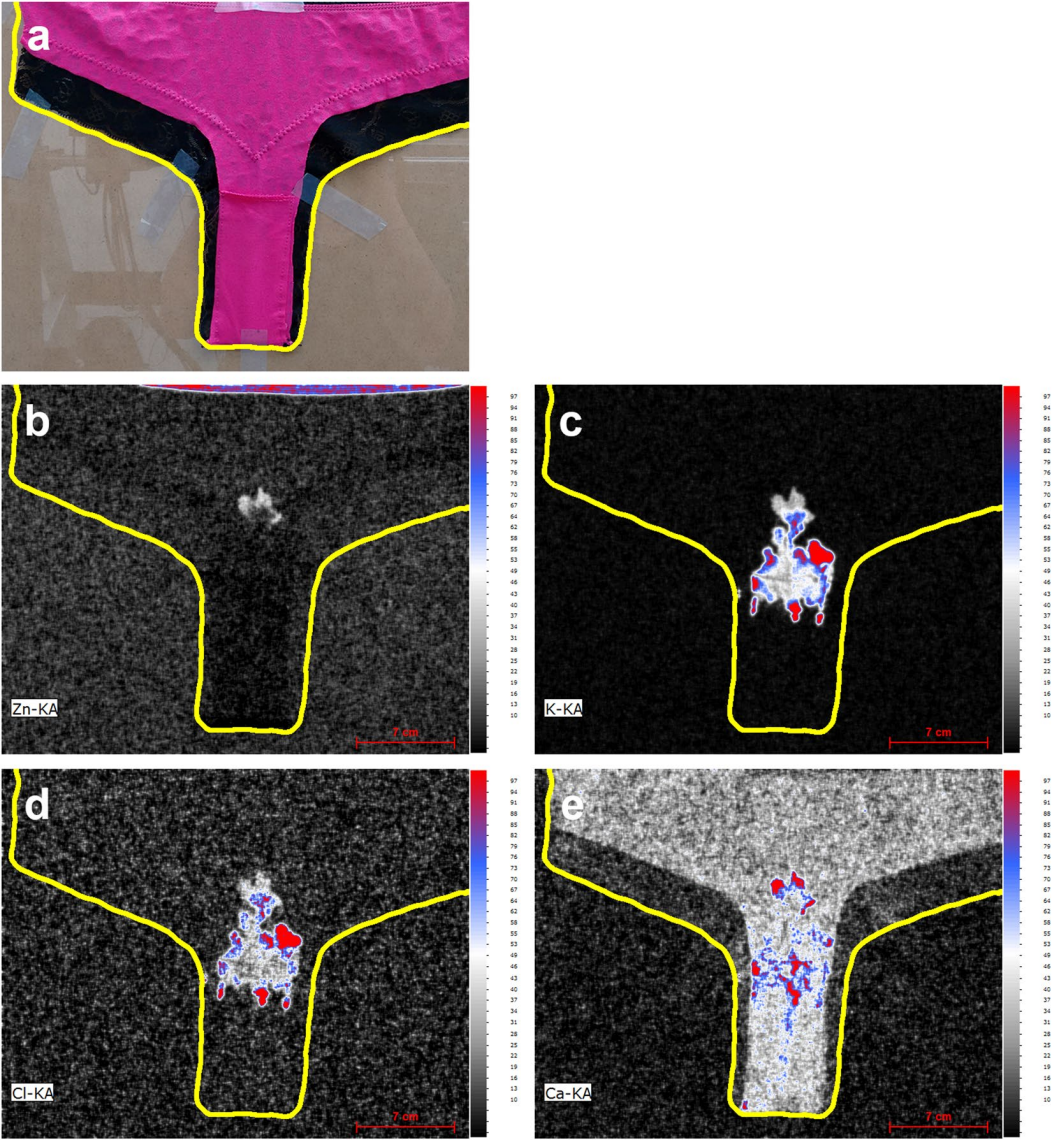
MA XRF elemental scans of Zn (**b**), K (**c**), Cl (**d**) and Ca (**e**) of female underwear (**a**) containing a semen and urine stain applied under controlled conditions (pixel scan time = 35 ms).

Despite this limitation with respect to stain size it was nonetheless expected that MA-XRF could be successfully applied for the detection and imaging of biological forensic traces. This was confirmed by the typical XRF spectra (given in Figure S3 of the Supplementary information) obtained for human biological stains under controlled conditions. Human blood can be detected with XRF through the elements K, Cl and Fe, semen through the elements K, Cl and Zn, saliva through the element K and urine and sweat through the elements K, Cl and Ca. Fe and Zn can be considered as marker elements to discriminate blood and semen from other biological matrices but the relative signals of the other elements were found to be highly consistent and can also serve as a source of information to establish the nature of the biological stain. Next, a number of proof-of-principle experiments were conducted to illustrate the forensic potential of MA-XRF imaging of biological stains. These experiments are based on scenarios involving victim clothing evidence items as encountered in actual case work but consisting of fabric types that are challenging for stain detection with conventional techniques. The first experiment involved the application of blood stain patterns (fingermark impression, impact, drop and expiration pattern) on a black cotton T-shirt. A photograph of the T-shirt and the corresponding XRF elemental maps for Fe, K and Cl are shown in Fig. 1. By combining spectral lines and using the relative abundance of the various elements, a maximum sensitivity and contrast of the blood stains versus the fabric background could be established. Although elemental sensitivity and fabric background levels vary, the results clearly illustrate how MA-XRF imaging can assist in detecting biological traces and direct sampling for further analysis such as DNA STR profiling. Additionally, the elemental images are of sufficient quality to forensically interpret patterns and spatial locations of the biological stains to assist in reconstruction and to test witness, victim and suspect statements. At these M6 scan settings (pixel size of 500 µm, pixel scan time of 35 ms and a total scan time of 17 hours) the image resolution is not high enough to establish trace characteristics such as fingermark patterns that would allow comparison against reference fingerprints (assuming that the impression contains such detail). The options and consequences of high resolution MA-XRF imaging will be discussed in more detail in the next section on gun shot residues and in the closing discussion. The results of a second experiment based on a sexual assault scenario are given in Fig. 2. In this case a partly mixed stain of urine (typically victim related) and semen (typically perpetrator related) was applied to brightly colored and fluorescent female underwear. XRF imaging of the stains of forensic interest was feasible through the elements Zn, K, Cl and Ca. As expected the element Zn is the most distinctive marker for semen but because Zn levels are relatively low in this biological fluid the overall sensitivity using the Zn signal is limited.

The results from the proof-of-principle experiments are promising, but to fully establish the forensic potential of XRF imaging of biological traces the selectivity of the associated elements needs to be investigated further. Marker elements such as Fe, Zn, K, Cl and Ca are inherently not very selective and their presence may be abundant in substrates of interest and in stains and residues without forensic relevance that may be present on clothing. First a broad collection of fabric types that are commonly used in modern clothing were screened with XRF for background element levels. An overview of the findings is presented in Table S2 of the Supplemental Information. Significant background levels of Zn were observed for two less common fabric types (imitation leather and chequered shear) whereas Fe was found on four test cloths including more commonly used fabric types such as black cotton, denim and wool. However, as is illustrated in Fig. 1 detection of blood stains is still feasible on a fabric containing relatively high elemental background levels as long as significant differences in elemental concentrations exist. The use of elemental ratios and combined elemental image maps could in such instances improve the performance. Interestingly, K and Cl were hardly observed for the fabrics tested whereas Ca was found on eight of the ten fabric types. The widespread presence of Ca is not surprising and can be expected for all washed clothing. Although background levels on substrates decrease the sensitivity it will not affect selectivity, *i.e*. lead to a false positive outcome. Such a false positive detection could however result from stains on fabric that contain the same marker elements but originate from other sources than human biological fluids. Such stains could arise from spilled food stuffs, cosmetics and other personal care products and work related materials like grease, paint and ink. With the assistance of Unilever R&D in Vlaardingen, The Netherlands, an overview was made of frequently occurring stains on Western European clothing. Specially prepared swatches containing collections of standardized stain formulations used by home and personal care companies to assess stain removal efficacy of detergent formulations were kindly provided by Unilever for this study. The stains were scanned with MA XRF and their elemental profiles were measured and compared against the elemental signatures obtained for the human biological matrices. The results of this comprehensive screening exercise are given in Table S4 in the Supplemental Information. Of the total of 43 stain formulations tested 70% contained K, 53% showed the presence of Fe and 40% tested positive for Ca and Cl and 14% for Zn. As expected the main elements used to detect and image human biological evidence with MA-XRF are also encountered in common stains on worn clothing. Hence it is clear that confirmation of the human biological matrix requires additional methods such as indicative tests, immuno assays and DNA STR profiling. This will be a normal cause of action after trace detection and hence limited selectivity is not too problematic as long as false positives do not occur frequently as this would result in a waste of additional forensic resources (*e.g*. biological forensic investigations not yielding useful DNA profiles). In this respect it is important to note that, despite the fact that many of the studied stains contain elements of forensic interest, the overall elemental profiles of most stains differ significantly from the reference profiles obtained for the human biological fluids. As is illustrated in Figures S4 and S5 in the Supplemental Information, most of the stains on the test swatch could be differentiated from human blood, semen, saliva, sweat and urine on the basis of their overall XRF elemental profile. However, the XRF profiles of red wine, grape juice and black coffee were found to be very similar to that of saliva whereas the elemental composition for the ketchup stain was comparable to the XRF data for sweat and urine. Several stains on the swatch contained the same elements as detected for blood but never with the same K:Fe ratio.

An additional test with respect to the usefulness of MA-XRF for forensic case work was undertaken to study the effect of the age of the traces. It is quite common that evidence items are investigated by forensic experts months and sometimes even years after the actual criminal activities occurred. Within the criminal justice system the time interval between the crime scene investigation and the investigation of the evidence at the forensic institute can span several weeks. Furthermore, cases sometimes involve the discovery of clandestine graves years after the criminal activities occurred. For the detection and imaging of aged biological traces, MA-XRF could be of added value because elemental signatures are not affected by oxidation states and are not expected to degrade. This was confirmed by the almost identical MA-XRF scans that were obtained for a fresh blood stain and the same stain after more than 4 months of ageing under ambient conditions (as shown in Figure S6 of the Supplementary Information). This feature of MA-XRF could be very useful when visualizing and interpreting stain patterns of aged and degraded biological stains on *e.g*. cold case evidence items.

In forensic casework, detected biological traces are sampled for DNA profiling. Thus, it is essential that detection techniques are non-invasive with respect to the genetic material in biological traces. To this end, the effect of the x-ray radiation required for MA-XRF imaging on the genetic material in human bloodstains was studied. STR DNA profiling was conducted on multiple human bloodstains unexposed to X-ray radiation, after regular MA-XRF imaging (single MA-XRF scan), and after extensive X-ray radiation exposure (the equivalent of ten consecutive MA-XRF scans). For all conditions full and high quality STR DNA profiles of the donor were obtained. As is illustrated in Figure S7 of the Supplementary Information, the amount of DNA sampled from the bloodstains is comparable irrespective of the received dose of X-ray radiation. These results confirm that the use of MA-XRF does not negatively affect the DNA profiling and that the technique can safely be used in a forensic setting.

### Elemental mapping and forensic interpretation of gunshot residues on clothing

The reconstruction of complex shooting incidents requires that a lot of information is obtained from the trace patterns formed during shooting. Multiple weapons can be involved and often various types of cartridges are retrieved from a crime scene. The clothing of a victim is an invaluable source of information that can contribute to the forensic interpretation of a shooting incident. In this study the use of MA-XRF for scanning a complete T-shirt and by doing so recording the full trace pattern of gunshot residue (GSR) was explored. The acquired data was then analyzed to answer forensic questions contributing to the reconstruction.

**Figure 3:**
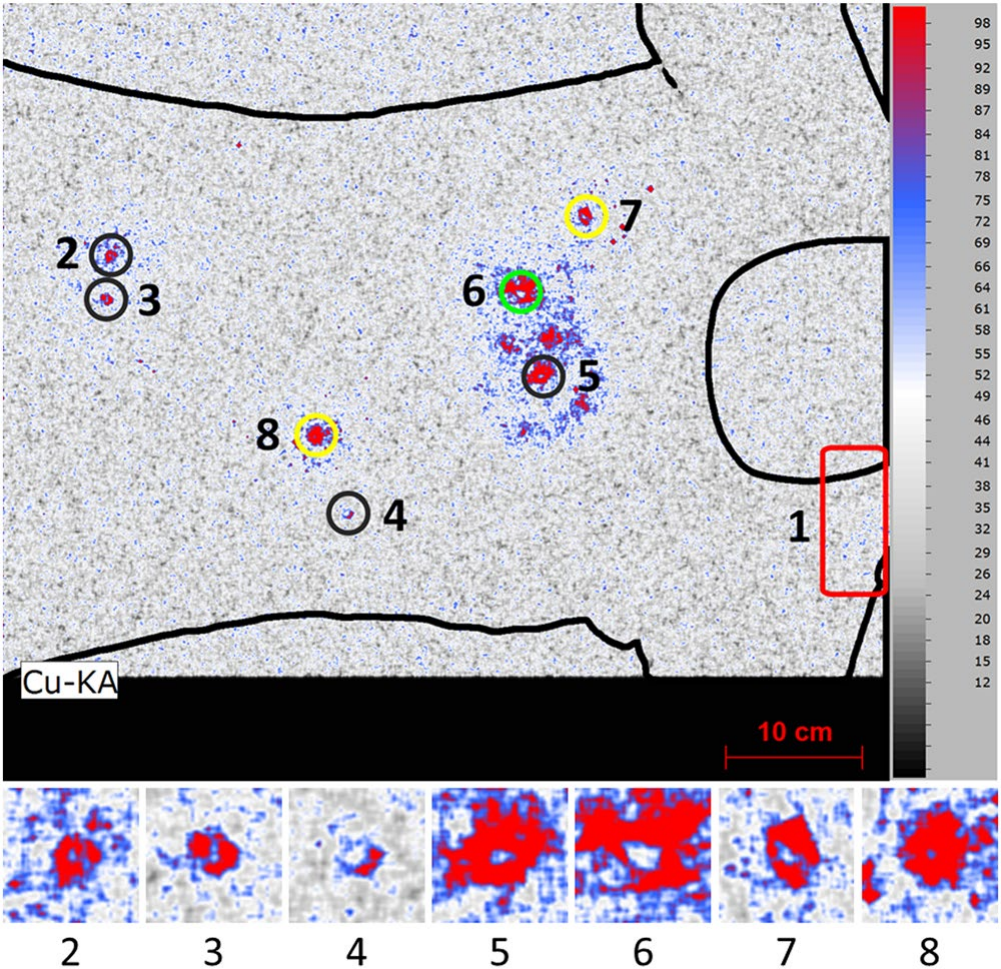
XRF elemental image of Cu indicating the presence of bullet holes. The various colored circles correspond to the different types of ammunition, *i.e*. CBC Magtech (black), Speer Lawman (green) and Makarov (yellow). The small image inserts show the details for each bullet hole (pixel scan time = 60 ms).

**Figure 4:**
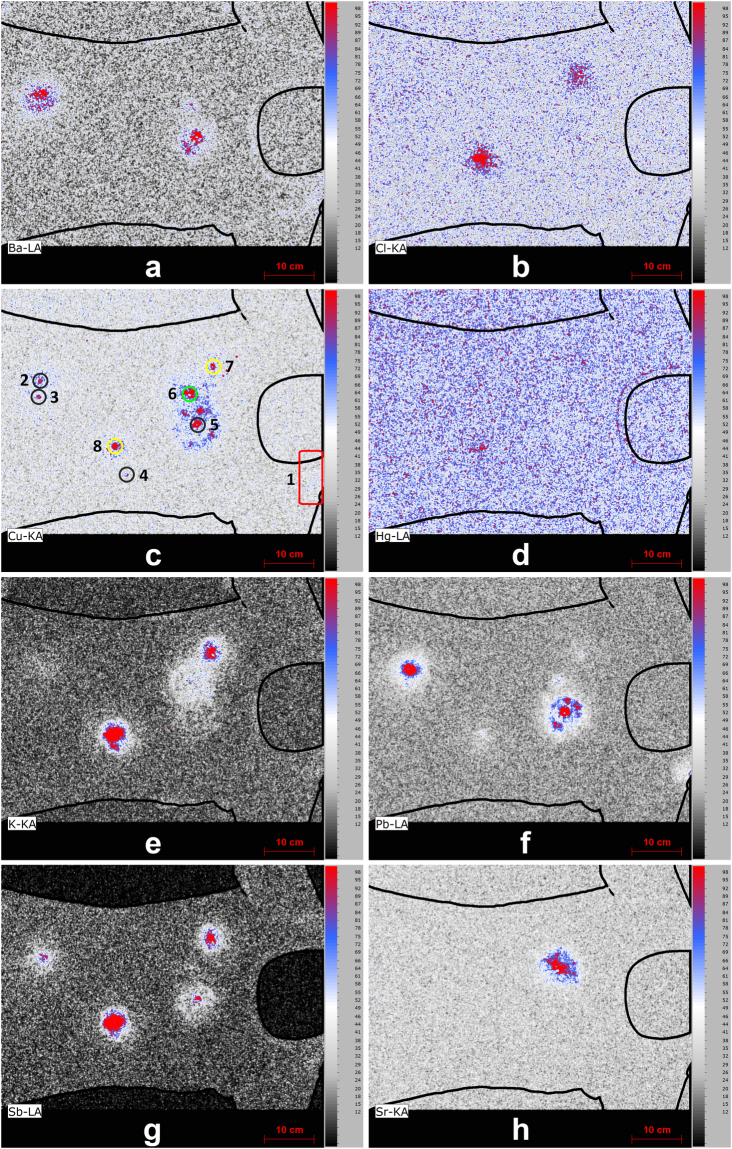
Characteristic elements that can used for chemical imaging and matching bullet holes with the various types of ammunition (see circles in the Cu image with CBC Magtech (black), Speer Lawman (green) and Makarov (yellow), pixel scan time = 60 ms, a = Ba, b = Cl, c = Cu, d = Hg, e = K, f = Pb, g = Sb and h = Sr).

The XRF elemental image of Cu proved to be an excellent starting point for the analysis as high concentrations of this element were found at spots corresponding to the location of bullet entrance holes, as is illustrated in Fig. 3. After identifying the prime locations of interest, further analysis can be undertaken. The influence of various types of ammunition on the elemental images around the bullet holes was explored. In these experiments three types of ammunition (*i.e*. CBC Magtech, Speer Lawman and Makarov) comprising different primer compositions were used. These types of ammunition with various primer compositions are expected to deposit different elements around the bullet hole. In Figure S8 of the Supplementary Information similar short (10 centimeter or shorter) shooting distances and similar angles of incidence (90 degrees with respect to the target material) are compared. From this comparison it becomes clear that although measurement conditions are far from optimal (*e.g*. overlapping trace patterns, ambient conditions suppressing the detection of low energy x-ray photons and the lack of optimized measurements conditions for each separate hole) sufficient elemental differences are revealed to be able to match the types of ammunition to specific bullet holes. Based on the spectra Pb, Cu, Cl, K, Sb, Ba, Sr and Hg were selected and plotted in the form of the elemental images depicted in Fig. 4.

Comparing these elemental images with trace patterns around the three shots reveals that in the case of Speer Lawman ammunition (shot 6) the element Sr is a solid indicator for the presence of such ammunition. Potassium can be used as an additional marker, but this element is also present in the Makarov ammunition (shot 8) and thus will be found around multiple bullet holes. In the case of Makarov ammunition, however, K is accompanied by Sb, Cl and to a lesser extent by Hg. The presence of Hg around the bullet hole is however difficult to detect in the elemental image. All aforementioned elements are absent in the CBC Magtech ammunition (shot 2), but in this ammunition Pb and Ba are present at major levels. Sb was found as well but this element is also detected for the Makarov ammunition.

As can be seen in Fig. 4, it was not possible to uncover elements characteristic for the various types of ammunition for all bullet holes. In this proof-of-principle experiment, this limitation is likely the result of large (*i.e*. 50 centimeter or more) shooting distances for some shots. At medium to long shooting distances less trace material will be deposited around the bullet hole as the converging gas cloud emerging from the barrel quickly dilutes. For these distances, the bullet wipe deposited by the bullet is therefore the only trace. For shooting distances exceeding 50 cm, more detailed investigations are necessary to determine the origin of the bullet hole. This can be achieved by a high resolution scan with longer acquisition times or by using other techniques such as SEM-EDS, as is done in present day casework.

A great benefit of using MA-XRF imaging compared to SEM-EDS analysis, however, is that the location of the trace elements can be taken into account. This is especially useful for neighboring bullet holes that show significant overlap of their GSR trace patterns. When using stubs for sampling in preparation of SEM-EDS one picks up particles related to the various bullet holes. The information regarding which particles belong to which bullet hole will be easily lost, whereas such data is maintained in the elemental images created by MA-XRF. In this respect the combined use of MA-XRF and SEM-EDS, where the trace pattern is first captured non-invasively with MA-XRF followed by sensitive, detailed SEM-EDS particle analysis after invasive stubbing, could be very useful in GSR case work.

In addition to linking bullet holes to specific types of ammunition, knowledge on the various elements found around the holes can be used to look for additional traces on a piece of evidence. In these experiments, increased concentrations of Pb and Ba were found in the area of the left shoulder. In this case, a close range shot (number 1) was fired just over the left shoulder of the T-shirt (mimicking a near-hit in a shooting incident). Such findings therefore can be used to check statements provided by witnesses, suspects or victims and can be used as input for determining sampling strategies.

As discussed in the introduction, previous XRF shooting distance studies have all focused on one bullet hole per scan. In the experiment described here, it is shown that it is possible to use the data acquired during the scan of a complete piece of evidence for multiple shooting distance estimations. The images in Figure S9 of the Supplementary Information represent an area of 15 × 15 centimeter around CBC Magtech bullet holes (shots 2, 3 and 4 and 5) created at four different distances (2.5 cm, 10 cm, 50 cm and 150 cm, respectively). These images show that short shooting distances can easily be recognized as high concentration regions directly around the bullet holes surrounded by lower concentration regions further away. For all elements, the distribution patterns fade with increasing shooting distances and eventually disappear almost completely in the images corresponding to 50 and 150 centimeter.

Besides information on the shooting distance, a closer look into the elemental images around shots 7 and 8 reveals a difference in symmetry around the bullet holes, as can be seen in Figure S10 of the Supplementary Information. These two shots with Makarov ammunition were both fired from a 10 centimeter distance but at an angle of incidence of 45° and 90°, respectively. It can be seen that the patterns of Sb and Cl – both of which are attributed to the cloud of materials leaving the barrel – are unevenly distributed around the bullet hole created under a 45° angle of incidence (shot 7). This effect is less pronounced in the Cu image which is more related to the traces from the bullet itself. Also a slight difference in intensities between the two shots is noticeable with more GSR being deposited at the 90° angle. This implies that interpretation of such elemental images should be done with great care as various parameters are of influence on the distribution and intensity of the final trace pattern.

**Figure 5:**
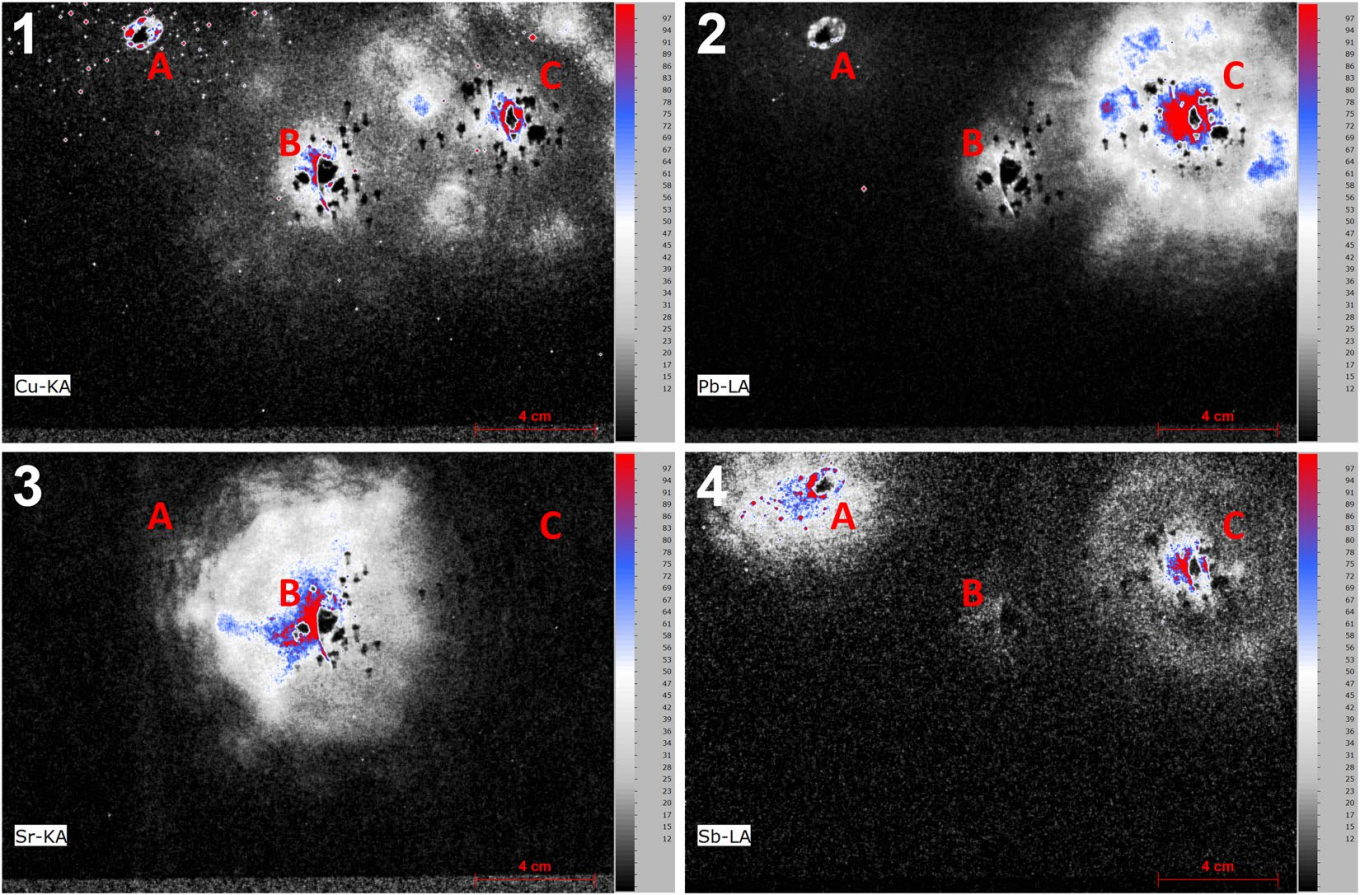
High resolution elemental images of the area in the middle of the T-shirt containing 3 shots; (**A**) Makarov ammunition fired from a Russian Makarov pistol at 10 centimeter and 45 degree angle of incidence with respect to the target material, (**B**) Speer Lawman ammunition fired from a Glock 17 pistol at 2.5 centimeter perpendicular to the target material and (**C**) CBC Magtech ammunition fired **prior** to shot B using similar conditions (pixel size = 150 µm, pixel scan time = 150 ms, 1 = Cu, 2 = Pb, 3 = Sr and 4 = Sb).

Next, the results of a high resolution scan containing the bullet holes located at the upper side of the T-shirt are discussed. From the elemental images in Fig. 5 it can be seen that bullet hole B produced by lead-free Speer Lawman ammunition (as shown by the presence of Sr) shows traces from Pb and Sb which are not supposed the be present^15^. In addition, the patterns are much smaller than the Sr pattern while no Sr was detected around bullet hole C. In this experiment, the presence of Pb and Sb can be explained by a memory effect, where residual materials originating from prior shots (here with CBC Magtech ammunition) are released when the weapon is fired consecutively. In casework, particular combinations of elements as shown here can be combined with other forensic investigations (*i.e*. when cartridges found at the crime scene are linked to one weapon) in order to determine the order of shooting. This might provide important legal insight if only one of several shots was fatal.

### Detection of concealed traces with XRF depth profiling

**Figure 6:**
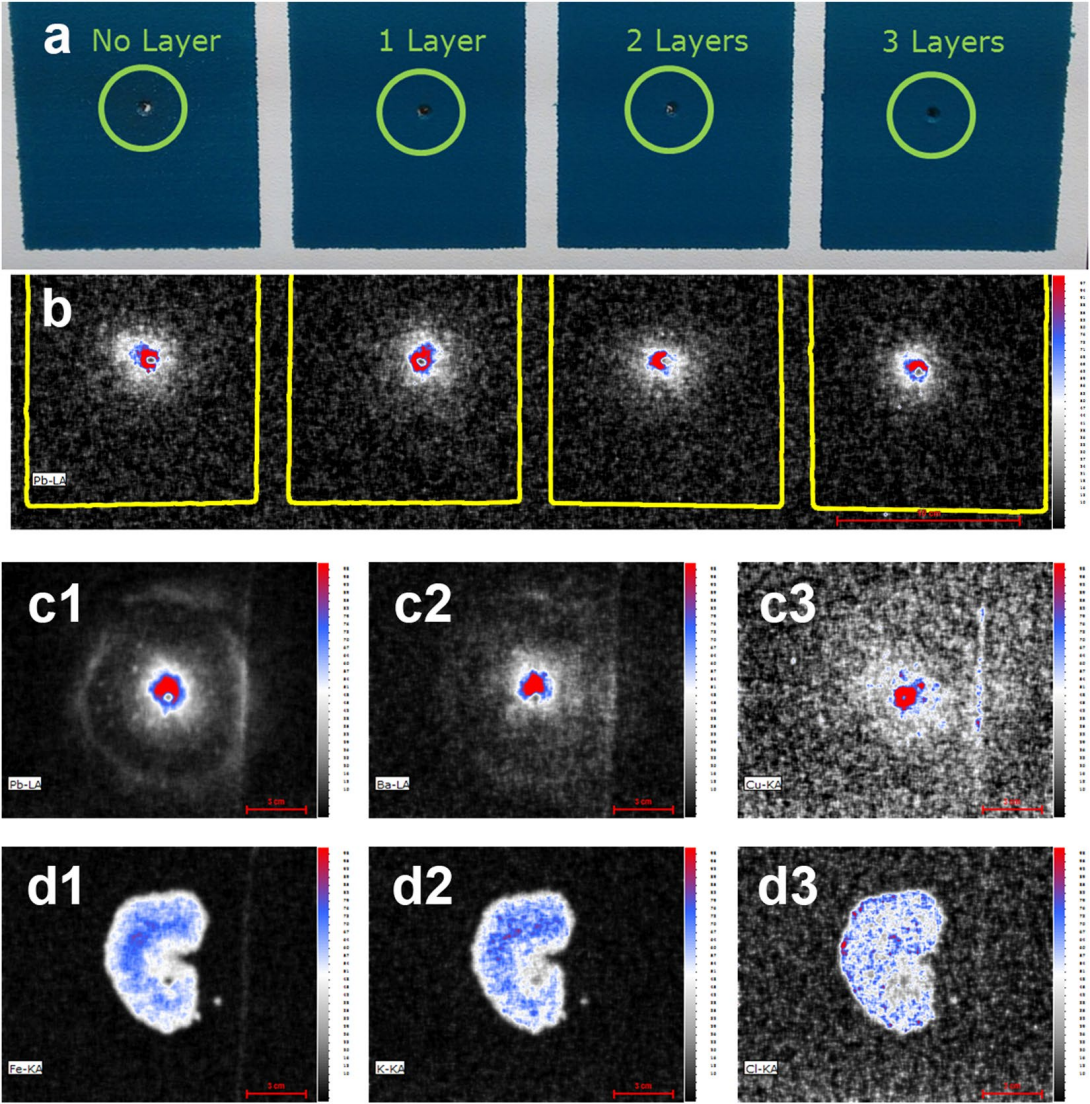
MA XRF Pb elemental image (**b**) of paint concealed gun shot residue on 15 × 15 cm plasterboard panels (**a**) and MA XRF elemental images of gun shot residues (Pb (**c1**), Ba (**c2**) and Cu (**c3**)) on a piece of black cotton covered with a blood stain (Fe (**d1**), K (**d2**) and Cl (**d3**)) of a few milliliters (CBC MAGtech 9mm PARA ammunition fired from 10 cm distance with a Glock 17 firearm).

MA-XRF can also be of use in criminal investigations when forensic experts are confronted with crime scenes where evidence is concealed either accidentally or on purpose. Such cases include locations were violent crimes including murders and manslaughter have taken place, after which the perpetrators have removed the bodies of the victims and have attempted to remove the evidence by cleaning and redecoration. For these special cases, application of MA-XRF at the crime scene could be valuable as it allows scene of crime officers to non-invasively scan areas for hidden forensic traces when the X-rays sufficiently penetrate the surface. To demonstrate the potential of MA-XRF for detection of concealed and hidden traces, two proof-of-principle experiments were conducted. The first experiment involved gunshot residues on a plasterboard concealed by multiple layers of strongly colored and concentrated wall paint. The results shown in Fig. 6a and b clearly illustrate that as long as the paint does not contain Pb, the GSR traces of lead containing ammunition can easily be detected through as much as three layers of paint by imaging the corresponding elemental signal. GSR traces on clothing of a victim can be fully or partly covered by blood as a result of the inflicted gun shoot wound. Also in this situation the depth profiling capabilities of MA-XRF can be exploited. This is illustrated in Fig. 6c and d showing the MA-XRF mapping of a GSR pattern that has been obscured by a large blood stain. The elemental patterns of Pb, Ba and Cu from the gun shot residue can be accurately imaged with MA-XRF in the presence of human whole blood. It should be noted that hidden traces can only be visualized in such a straight forward manner when the marker elements are not present in the material covering the stain of interest. If this is the case, visualization will be much more difficult but might still be possible with the use of spectral deconvolution or other data processing methods.

## Discussion and Conclusion

This study clearly shows the potential of MA-XRF imaging for forensic science. With the new generation of XRF imaging instruments such as the M4 Tornado and M6 Jetstream, elemental mapping of traces of forensic interest on various surfaces over substantial areas has become feasible. Benefits of XRF imaging in criminal investigations include the unique elemental contrast to detect and image trace patterns, the non-invasive nature of the imaging and the possibility of depth profiling which can reveal concealed evidence. However, for the robust and wide-spread use of XRF imaging in forensic case work several challenges still exist. The first challenge is to make the imaging equipment ‘*fit-for-forensic-purpose*’. Further optimization of the scan time versus resolution is also part of this challenge. Ideally evidence items could be studied at the forensic laboratory or police facility in a spectral bench setup combining expert inspection, photography and techniques such as hyperspectral, spectroscopic and XRF imaging. Such scans should be fast enough to provide results in a reasonable time frame to direct further investigation. On the basis of low resolution data, areas of specific interest could then be scanned at high resolution to retrieve as much information as possible from the forensic traces. The recorded spectral data and images should automatically be included in the case file. The semi-mobile application directly at the crime scene also has its specific requirements. In this case the XRF scanner should be sufficiently flexible to accommodate different surfaces to be scanned under different angles and positions. At the crime scene the amount of time available for the investigation is usually limited and hence the use of MA-XRF has to be even more effective and efficient.

The second challenge is to provide sufficient transparency and reproducibility in data handling and visualization. Especially when constructing (multi) elemental maps in which colors indicate the presence of an element and the intensity of the color is a measure for the amount present, the forensic expert should be able to clearly explain the nature of the image and the way it was created. Ideally, raw XRF data could be exported and processed with open source software so that the data processing and image creation are fully reproducible, understood, controlled and documented. However, the software packages used in this work are part of the instrumentation and are not open-source. Although the instrument software provides powerful options for data processing, the way that images are created and color maps are established are not entirely understood. Contrast and color intensity are relative and are determined by the overall presence of the element of interest. Hence the visualization of forensic traces is affected by background levels and additional stains and patterns (this is illustrated in Figure S11 of the Supplementary Information). We feel that providing options to set absolute or cut-off levels for color intensity would provide more flexibility and clarity. Even better would be the option to use calibration data to image elemental surface concentrations instead of detector signal. In this way also differences in color intensity between elements become meaningful.

The third and final challenge is to ensure that MA XRF imaging meets the quality requirements and is sufficiently validated to be used in actual forensic case work. Accreditation (*e.g*, according to the ISO17025 standard) requires an extensive validation study in which also the reproducibility and repeatability of the elemental imaging has to be established and documented. Such a comprehensive quality assessment was not included in the current study but a few experiments were performed to obtain an indication of the reproducibility of the μ-XRF data as obtained on the M4 instrument. For six blood stains (three stains each from two donors) ten consecutive point measurements were conducted yielding a relative standard deviation in the range of 2.0–2.8% for the intensity of the Fe-Kα line at 6.4 keV (details are shown in Table S4 of the Supplementary Information). As similar characteristics are expected for the M6 set-up^1^, current instrumentation for XRF imaging seems sufficiently robust and mature for validated and accredited application in forensic case work.

## Methods

### Materials, Substrates and Standards

Fabric samples were obtained by purchasing garments consisting of black cotton, white cotton, dark chequered cotton, brightly chequered shear, synthetic fabric (65% cotton, 30% polyester, and 5% elastane), tracksuit, denim, imitation leather and outer layer of a jacket. The majority of the fabrics were machine washed prior to analysis. For the proof-of-principle experiments black cotton T-shirts consisting of 95% cotton and 5% elastane were purchased at the Dutch convenience store Zeeman. Female underwear was purchased at the same store. Two white cotton multi stain swatches were provided by Unilever with in total 43 standardized stains that represent frequently encountered soiling on worn consumer clothing. The circular stains had a diameter of 2.6 cm. For the evidence concealment experiments 15 × 15 cm plasterboard plates with a thickness of 1 cm were used. The plates were painted with matted petrol blue wall paint (EAN = 5400107305094, Perfection). To establish the XRF calibration curves and detection limits for Fe, Zn and Pb standard solutions of 10.000 ± 30 µg/mL from Peak Performance were used (Fe Lot no 06B021, Zn Lot no 10G103 and Pb Lot no 10D176). Dilution series were prepared in demi water with final concentrations in the range of 10–10.000 μg/ml. Of the calibration solutions a volume of 670 ± 28 μl was accurately and evenly applied to a white cotton substrate leading to calibration spots with an average diameter of 2.6 cm corresponding to an area of 5.3 cm^2^.

### Sample preparation – Human Biological Traces

The body fluids used in this study were donated by healthy NFI co-workers who volunteered to provide samples for this study. The samples were obtained in accordance with the guidelines of the Crime Scene R&D unit of the NFI and after informed consent of the donors. Whole blood samples were stored in sodium citrate containers and stored in the refrigerator until used. For some traces whole blood was directly applied after a finger prick. Semen samples were stored in the refrigerator and used as such. Saliva, sweat and urine samples were directly deposited on the fabric after donation. The volume of the applied biological stains was in the range of 50–500 µL. In the proof-of-principle experiment four different types of blood stain patterns were produced to mimic a bloody nose drip, a bloody fingermark, expirated blood and an impact pattern. When handling and applying the biological samples special safety precautions were taken to prevent direct contact.

### Sample preparation – Gun Shot Residues

To prepare the samples with GSR patterns shooting trials were conducted at the firing range of the Netherlands Forensic Institute by forensic firearms experts and under strict safety protocols. Two semi-automatic firearms were used in the trials, a Makarov (PM) type pistol 1983 and a Glock 17 in combination with three different brands of ammunition, *i.e*. CBC MAGtech 9 mm PARA, Speer Lawman 9 mm Parabellum clean fire and 9 mm Makarov. With the exception of a special trial to investigate GSR carry over, prior to sample production 6 shots were fired to minimize memory effects. Shots were fired at different angles and different areas at shooting distances in the range of 2.5–150 cm. Most test firings were conducted at a shooting distance of 10 cm. An overview of all details regarding the shots on the T-shirt is given in Table S3 of the Supplementary Information. The concealed GSR traces were prepared by firing CBC MAGtech 9 mm PARA with a Glock 17 from a distance of 10 cm on four 15 × 15 cm plasterboard plates painted with matted petrol blue wall paints. Subsequently, three of the GSR traces on the plates were covered with 1, 2, or 3 layers of the same paint before the sample was analysed with XRF.

### XRF scanning

XRF scanning experiments were conducted on the M4 Tornado and the M6 Jetstream from Bruker. The M4 instrument can scan samples with dimensions up to 14 × 17 cm. The scanner uses an MCB 50–0.7 G Rh X-ray tube with a 0.2 mm Be window for element excitation. The X-ray tube was set at the maximum voltage of 50 kV with a corresponding current of 700 µA. Measurements were performed under ambient conditions. The system’s collimator produces an ellipse shaped spot size of 2.3 × 3.0 mm. The M4 uses the 30 mm^2^ Bruker Nano GmbH XFlash 430 – PA detector with an energy resolution <145 eV for Mn Kα at 300,000 cps. Unless stated otherwise, the measurements have been performed with a pixel size of 500 µm and a pixel scan time of 100 ms.

The M6 instrument can map areas of 80 × 60 cm in a single scan but larger area images can be constructed by combining elemental images. The system is equipped with a Rh-target microfocus X-ray tube that was set at the maximum excitation settings of 50 kV with a corresponding current of 600 µA. Scanning has to be performed under ambient conditions. The M6 uses polycapillary optics with a variable spot size in the range of 150–860 μm. A typical working distance of 1 cm results in a spot of approximately 350 μm. Measurements were performed with a pixel size of 500 µm, a pixel scan time of 100 ms unless specified differently. The M6 Jetstream uses a 30 mm^2^ XFlash Silicon Drift Detector with an energy resolution <145 eV for Mn Kα.

### Data processing

All data sets were analysed with the software package from the M4 Tornado (M4 Tornado v1.3) using the deconvolution and smoothing options to increase the elemental contrast in the mappings.

### DNA extraction and Nuclear DNA amplification

For the DNA extraction 20 bloodstains of one donor were deposited onto a white cotton T-shirt and divided into 3 groups; 5 bloodstains were each scanned once using regular settings of the MA-XRF, 5 bloodstains were scanned 10 times and the remaining 10 bloodstains were not scanned. After this, all complete bloodstains were cut out and DNA extractions were performed using the QIAamp DNA Mini Kit and the manufacturer’s instructions. To elute the DNA the following protocol was performed twice: 50 μl of pre-heated 25% AE buffer, an incubation time of two minutes at 70 °C and a centrifuging step of 1 min at 8000 rmp. The Alu repeat system was used with adaptions for determining the Human- specific genomic DNA concentrations of the bloodstains as described by Lindenbergh *et al*.^22^. The AmpFℓSTR®NGM™PCR Amplification Kit (Thermo Fisher Scientific, Foster City, Texas, USA (Applied Biosystems (AB)) was used for DNA profiling. Capillary electrophoresis with an ABI Prism 3130xl Genetic Analyzer ™ (AB) was used for the detection of PCR products. Genemapper ID-X version 1.1.1 (AB) was used for profile analysis with a detection threshold of 50 relative fluorescence units (RFU)^23^.

### Data Availability

The datasets generated and analyzed during the current study are available from the corresponding author on reasonable request. Details of the DNA STR profile of the anonymous donor at the Netherlands Forensic Institute cannot be made available because of privacy concerns.

## Electronic supplementary material

Supplementary Information
