# Supplementary material for: Large area imaging of forensic evidence with MA-XRF
Source: Sci Rep. 2017 Nov 8;7:15056. doi: 10.1038/s41598-017-15468-5 (PMC5678136; doi:10.1038/s41598-017-15468-5)
Supplement: Supplementary file 1 — Supplementary Information [file 41598_2017_15468_MOESM1_ESM.pdf]

Large area imaging of forensic evidence with MA-XRF

Kirsten Langstraat[1], Alwin Knijnenberg[1], Gerda Edelman[1], Linda van de Merwe[1], Annelies van Loon[2], Joris Dik[3], Arian van Asten[1,4,5]

[1]Netherlands Forensic Institute, P.O. Box 24044, 2490 AA The Hague, The Netherlands

[2]Rijksmuseum, P.O. Box 74888, 1070 DN Amsterdam, The Netherlands

[3]Delft University of Technology, Materials Science and Engineering, P.O. Box 5, 2600 AA Delft, The Netherlands

[4]University of Amsterdam, Faculty of Science, Van 't Hoff Institute for Molecular Sciences, P.O. Box 94157, 1090 GD Amsterdam, The Netherlands

[5]CLHC, Amsterdam Center for Forensic Science and Medicine, P.O. Box 94157, 1090 GD Amsterdam, The Netherlands

Correspondence and requests should be addressed to : Arian van Asten (a.van.asten@nfi.minvenj.nl)

Supplementary Information

Table S1 Typical levels of potential XRF marker elements in human biological fluids

(In bold : fluid marker element)

Sample	Element	Typical Level [μg/ml]	References
Blood	**Fe**	500	[SI.1]
	K	2000	[SI.1]
	Cl	3000	[SI.2]
Semen	**Zn**	150	[SI.3], [SI.4]
	K	1000	[SI.3], [SI.5]
	Cl	1500	[SI.3]
Saliva	K	1000	[SI.6]
Sweat	K	200	[SI.7], [SI.8]
	Cl	1500	[SI.8]
Urine	K	2000	[SI.2]
	Cl	3500	[SI.2]

Figure S1 Calibration curves for the elements Fe (a), Zn (b) and Pb (c)

(A volume of 670 ± 28 µl of calibration solution was applied on white cotton on an area of 5.3 cm^2, instrument : M4)

a)

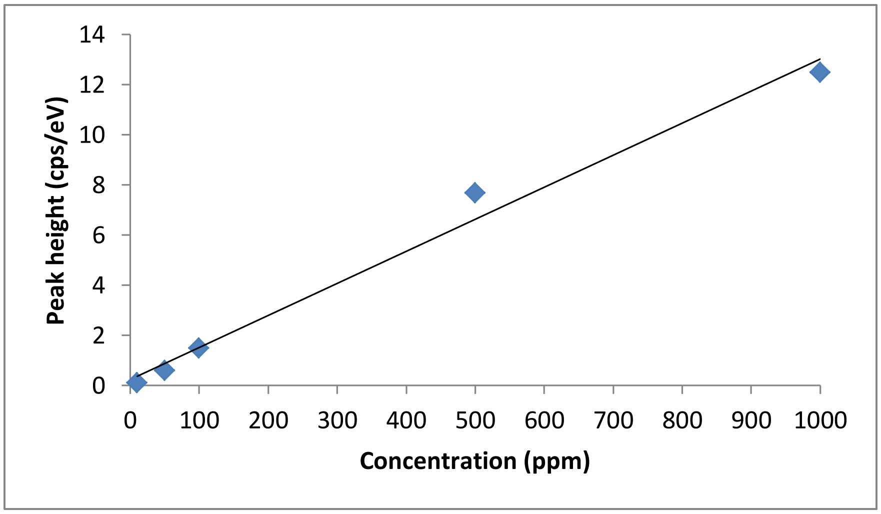

b)

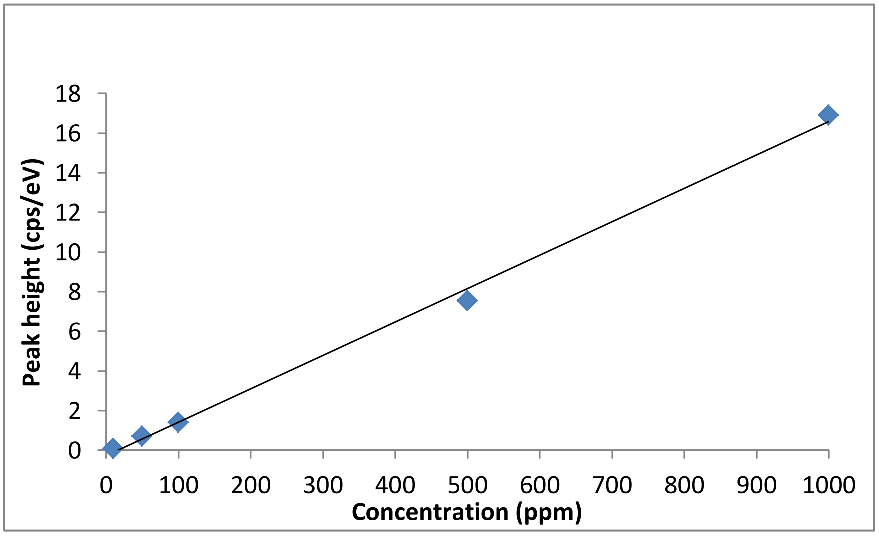

c)

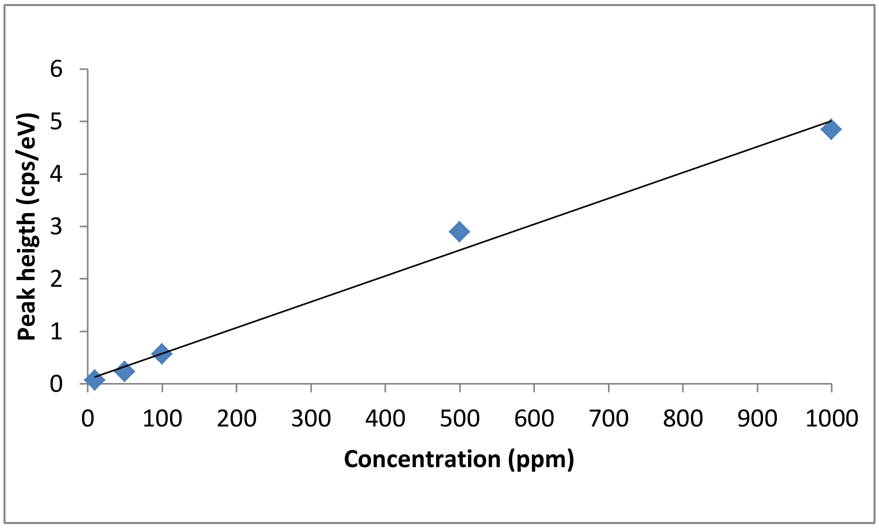

Figure S2 Effect of XRF scan area on Fe (a) and Zn (b) sensitivity and noise and Zn noise band (4xSD in cps/eV) as function of scan area/acquisition time (c) (A volume of 670 ± 28 µl of the 100 ppm calibration solution was applied on white cotton on an area of 5.3 cm^2, instrument : M4, pixel scan time = 10 ms).

a)

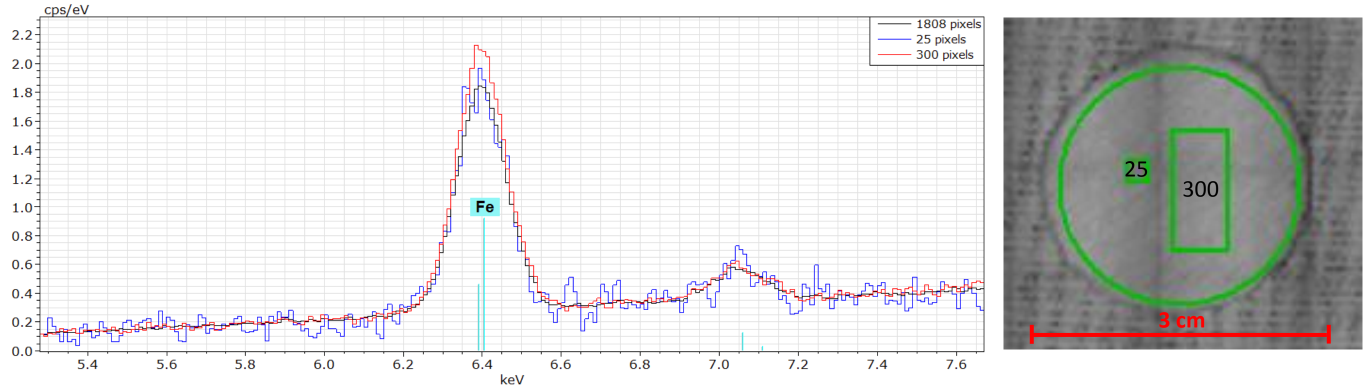

b)

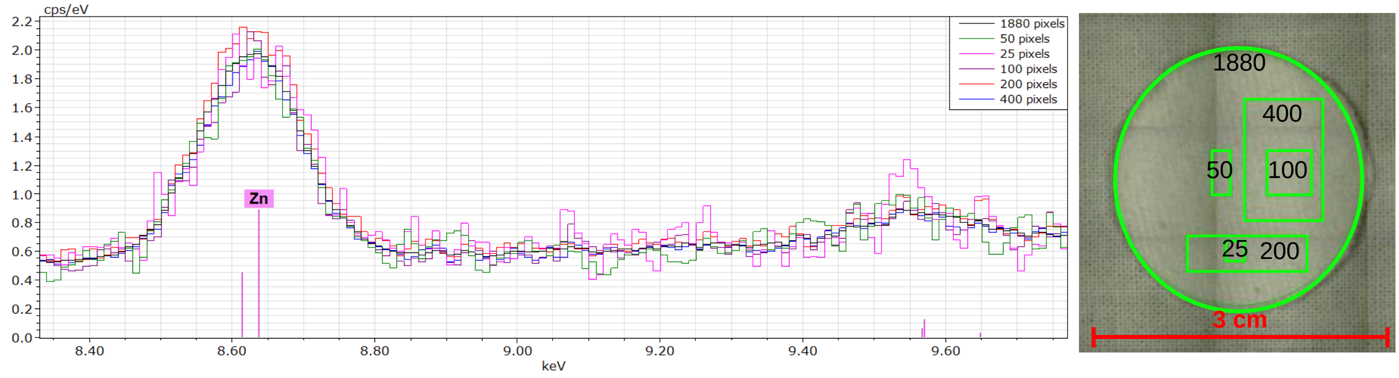

c)

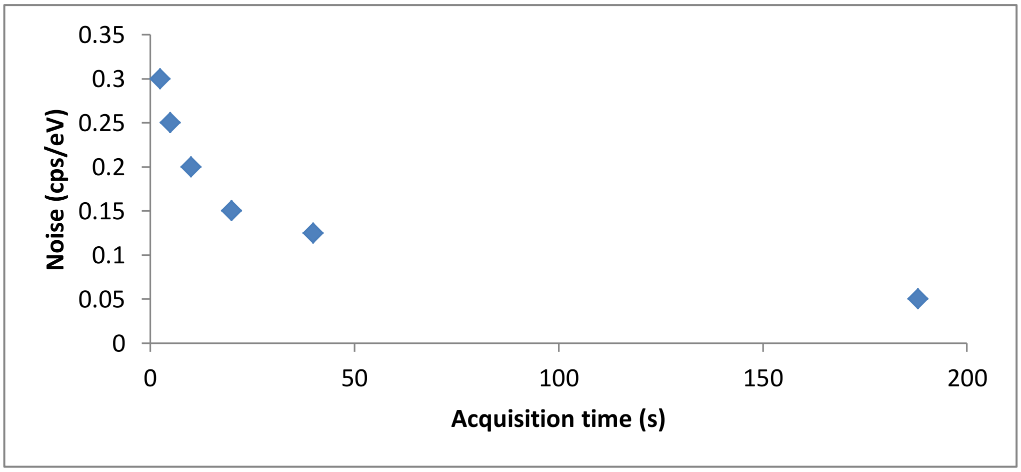

Figure S3 Typical XRF spectra for stains of human blood (a), semen (b), saliva (c), sweat (d) and urine (e) on white cotton . The consistent presence of Zr, Rh and Ar was due to external sources (respectively, the collimator, the X-ray tube and ambient air).

(Variable volumes (~0.5-1 ml) of human body fluid were directly applied on white cotton, instrument : M4)

a)

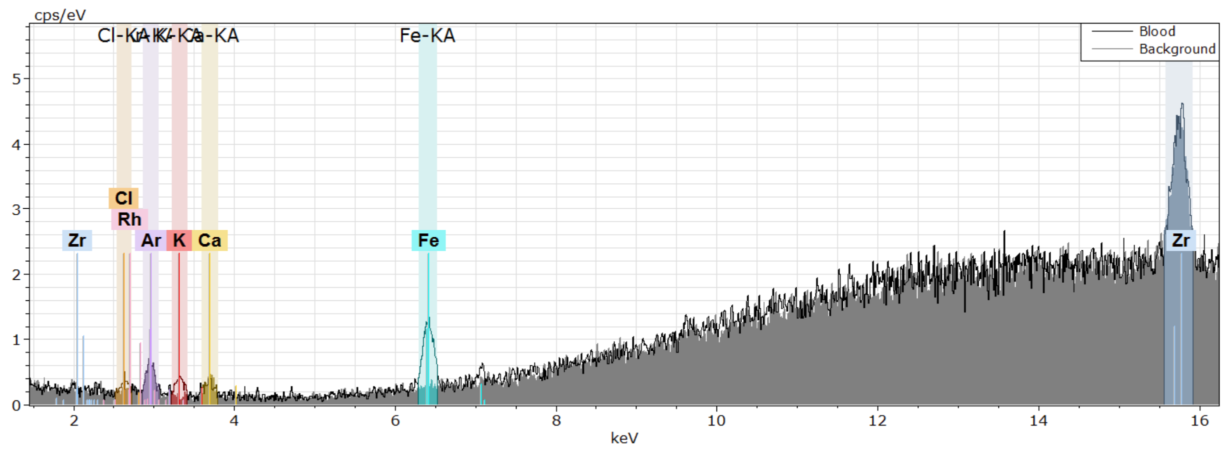

b)

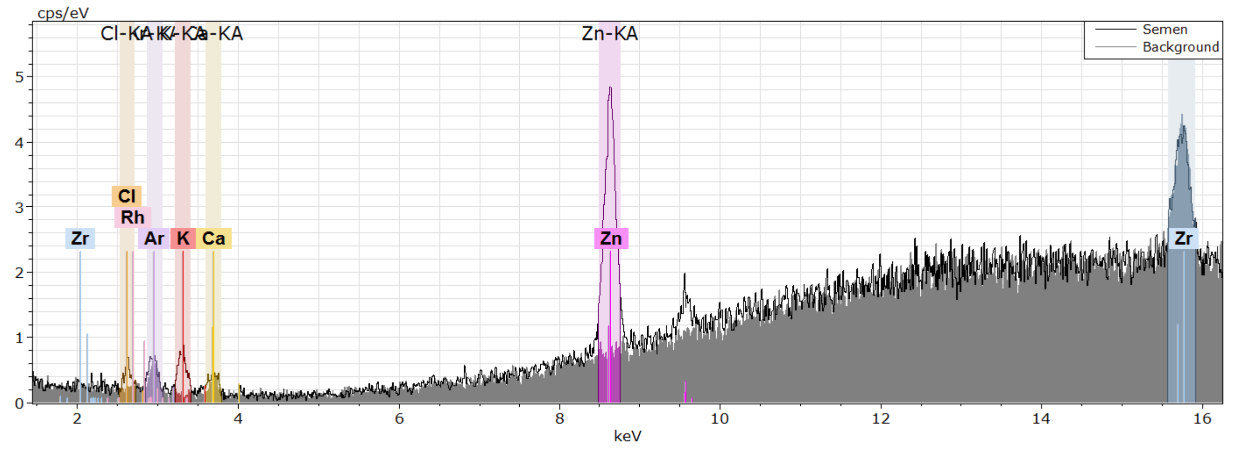

c)

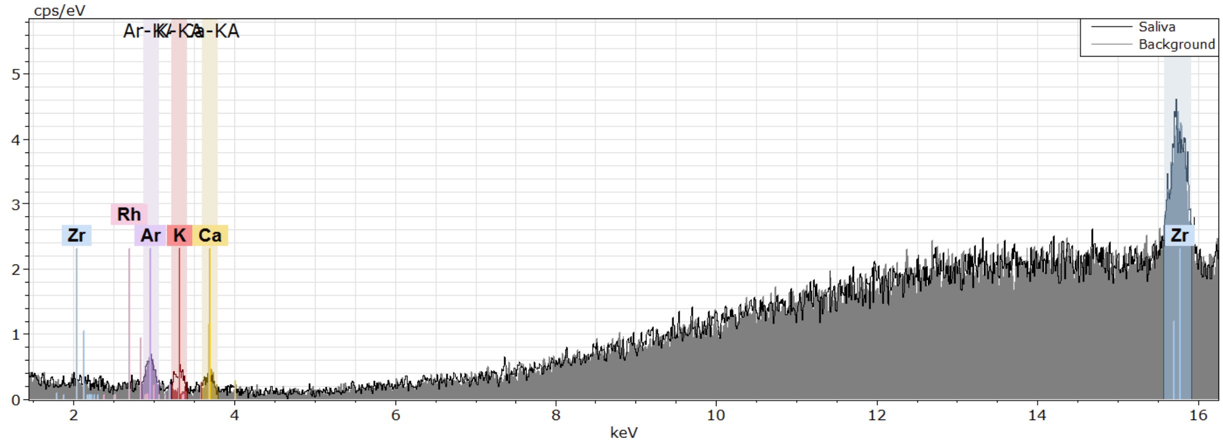
cps/eV
Saliva
Background
Zr
Rh
Ar
K
Ca
Zr
keV

d)

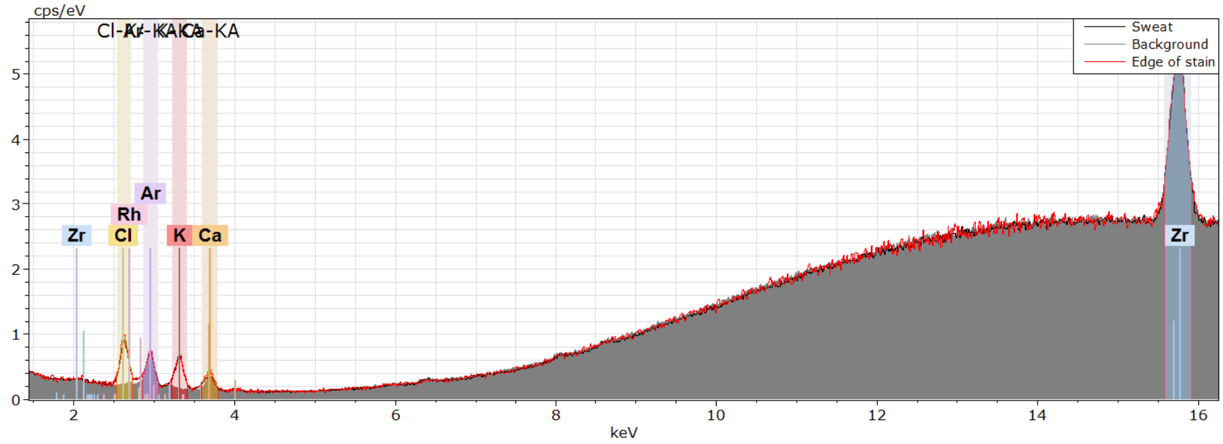
cps/eV
Sweat
Background
Edge of stain
Ar
Rh
Zr
Cl
K
Ca
Zr
keV

e)

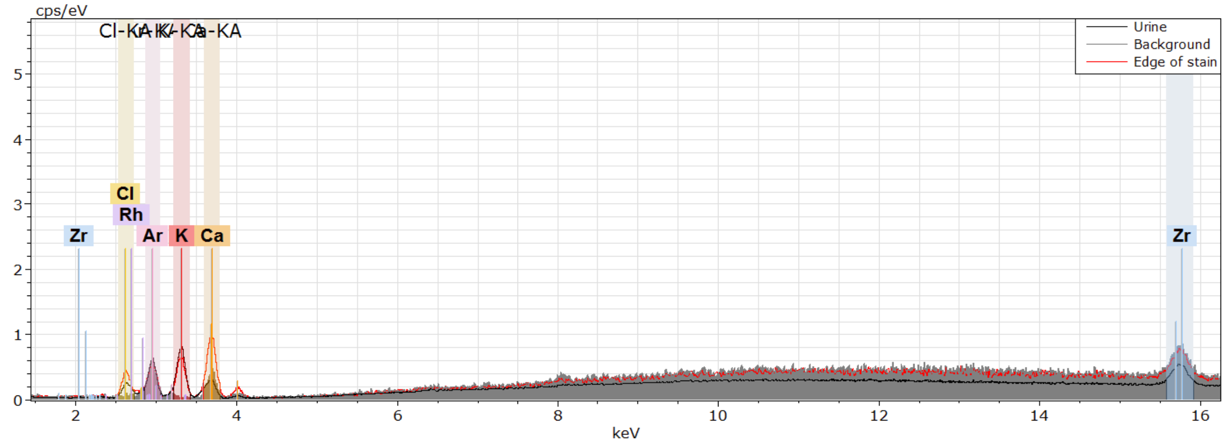
cps/eV
Urine
Background
Edge of stain
Cl
Rh
Zr
Ar
K
Ca
Zr
keV

Table S2 Elements observed with XRF scanning for various fabric types.

Instrument : M4 (red = low, yellow = medium, green = high abundance)

Type of fabric	Visual image	Elements detected
Black cotton	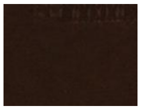	Ca, Fe
White cotton	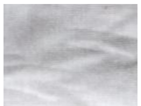	Ca
Dark chequered cotton	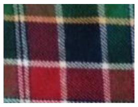	Ca
Brightly chequered shear	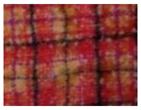	S, Ca, Cr, Zn
Synthetic (65% cotton, 30% polyester, 5% elastane)	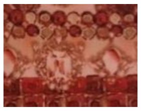	Ti, Cl
Tracksuit	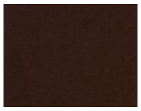	Ti, Br
Denim	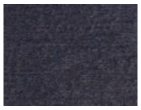	Ca, Ti, Fe
Imitation leather	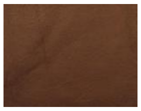	Si, S, Ca, Cr, Fe, Zn
Shiny outer layer of a jacket	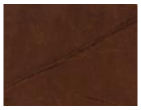	Ca, Ti, Br
Wool	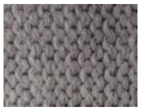	S, Ca, Ti, Fe, Cu, Br

Table S3 Elements observed with XRF scanning for stains frequently encountered on clothing in Western Europe. Instrument : M4.

(red = low, yellow = medium, green = high abundance)

Stain	Visual image	Elements detected
HP Brown Sauce	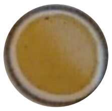	Cl, K, Ca, Fe
Ragu/Sunflower Oil	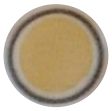	Cl, K, Ca, Fe
Blue Poster Paint – Color & Co. Ready Mix	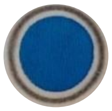	Ca, Fe, Cu, Br
Heinz Tomato Ketchup	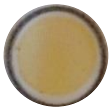	Cl, K, Ca
Face Make-up 2 – Max Factor Lasting Performance 109 – Natural Bronze	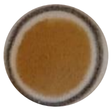	Si, Cl, Ti, Fe
Green Curry (Mae Ploy) + Ranuka Tinned Coconut Cream	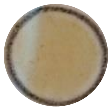	S, Cl, K, Mn, Fe
Red Pottery Clay 2:1 water : clay dry fabric	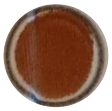	Si, K, Ti, Cr, Mn, Fe, Ni, Zn, Rb, Sr
Hipp Organic Carrot & Potato Baby Food (pureed carrot) Double Application	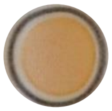	Cl, K, Ca, Fe
PG Tips – White Tea	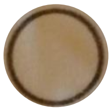	K, Mn
Grape Juice (Welches Black Grape Juice)	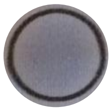	K
Cocoa Milk (Droste) ex VI	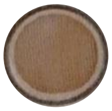	S, Cl, K, Ca, Fe

Snickers Chocolate Milk Shake	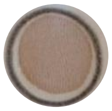	P, S, Cl, K, Ca
Black Coffee (Lyons Filter Coffee)	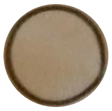	K
Mechanical Grease	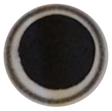	Ti, Fe, Zn, Mo
Gravy (Vlaar)	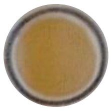	Cl, K
Sebum	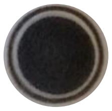	-
Cooked Beef Fat	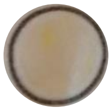	-
Beef Patty Dripping	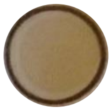	P, S, Cl, K, Fe, Zn
Red Pepper/Oil/Water	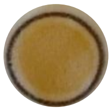	K, Fe
Dende Oil Unheated (Satellite ex Brazil)	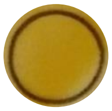	-
Quink Water Based Fountain pen Ink	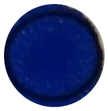	S
Tomato (Pomarola)/Sunflower Oil	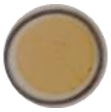	Cl, K, Ca, Fe
Rimmel Lipstick Coral in Gold	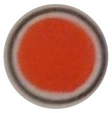	Si, K, Ti, Fe
Rubbed in Grass	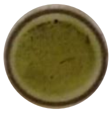	S, Cl, K, Ca, Fe, Zn

Chocolate Ice-cream (premium) (Haagen Dazs)	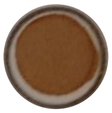	Cl, K, Ca, Fe
Blackberry whole fruit (ASDA in unsweetened apple juice)	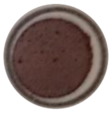	K, Ca, Fe
Choc Ice Cream (Economy) (ASDA)	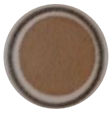	Cl, K, Ca, Fe
Kiwi Black Shoe Polish (liquid polish)	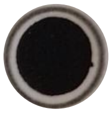	Ca, Fe
Gravy (instant)	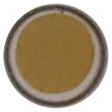	Cl
1:1 Garden Soil : Water	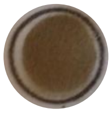	Si, K, Ca, Ti, Cr, Mn, Fe, Zn
1:1 Indian Red soil clay	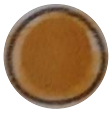	Si, K, Ti, Cr, Mn, Fe
Yellow Pottery Clay 2:1 water:clay dry fabric	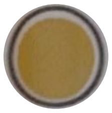	Si, K, Ti, Cr, Mn, Fe, Ni, Zn, Rb, Sr
Choc Pudding (Heinz)	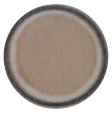	Cl, K, Ca
Yellow Curry Rajah Mild and spicy)	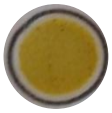	K, Ca, Fe
Red Curry (Osman)	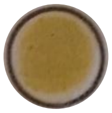	K, Ca, Fe
Face Make-up 1 (Rimmel stay Beige)	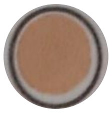	Si, Cl, Ti, Fe
Blackcurrant juice (Eden)	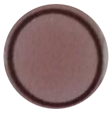	K

Black Tea	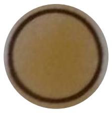	K, Mn
Red wine	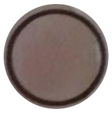	K
Lard + violet dye	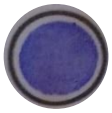	-
Annatto/oil	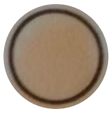	-
Cooking Oil + violet dye (Sunflower Oil)	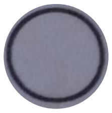	-
Mascara	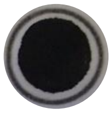	K, Fe

Figure S4 Elemental profiles of some stains from Table S3 containing Fe (b = Rimmel lipstick ‘coral in gold’, c = shoe polish and d = Rimmel face make-up ‘stay Beige’) compared to the XRF profile for human blood (a). Instrument : M4.

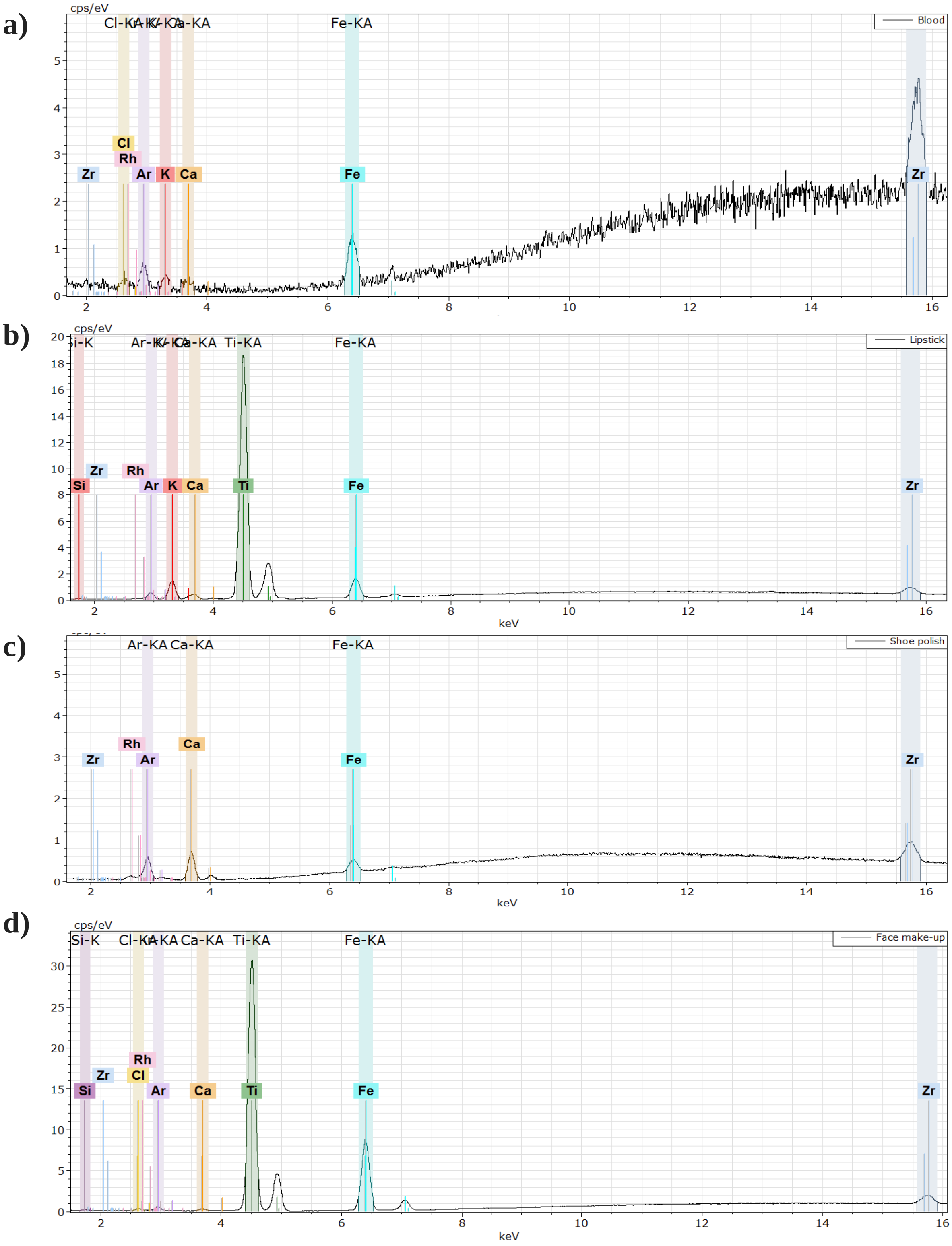

Figure S5 Elemental profiles of some stains from Table S3 containing Zn (b = beef, patty dripping, c = rubbed in grass and d = mechanical grease) compared to the XRF profile for human seminal fluid (a). Instrument : M4.

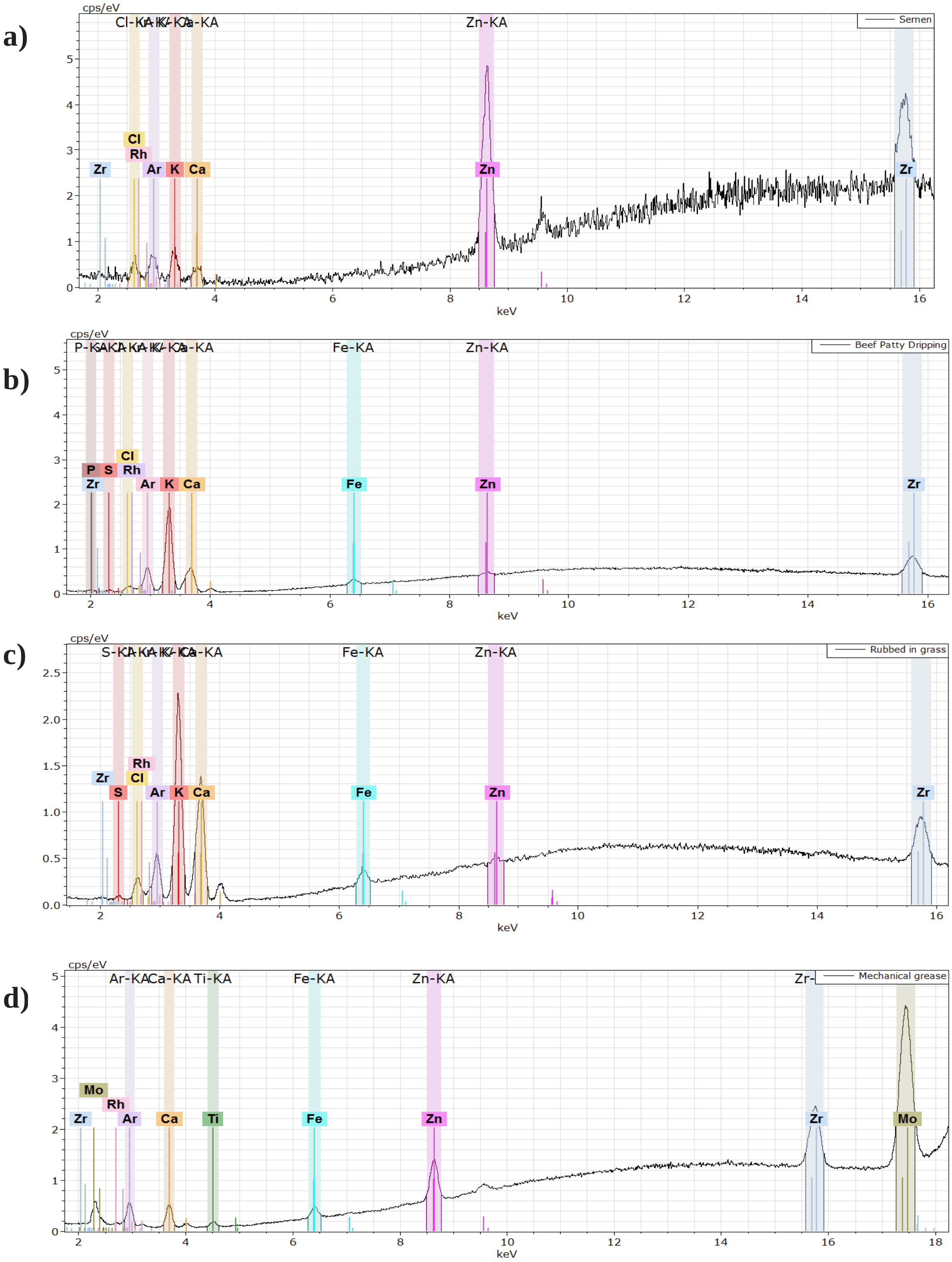

Figure S6 Elemental XRF profile of a fresh (a) and 4.5 month old (b) blood stain (Storage conditions: ambient, unmonitored). Instrument : M4

a)

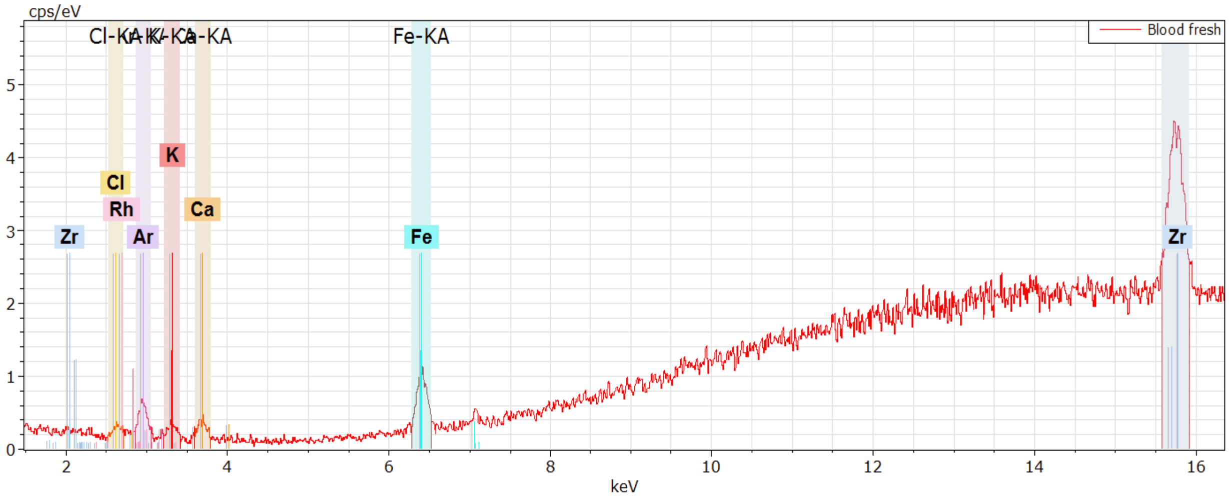

b)

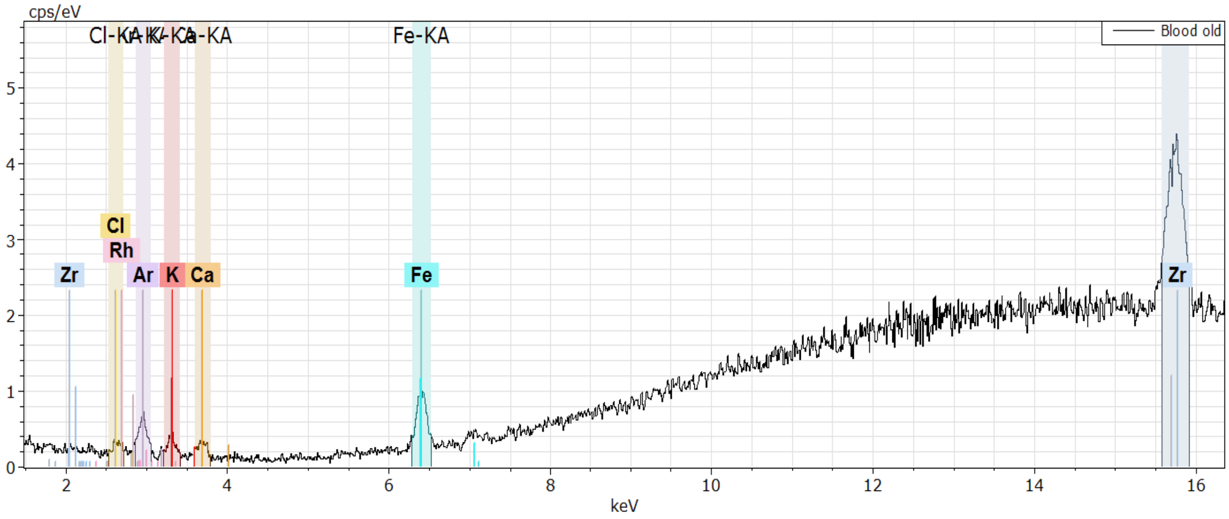

Figure S7 Amount of DNA in extracts of sampled blood stains after 0, 1 or 10 times MA-XRF scanning.

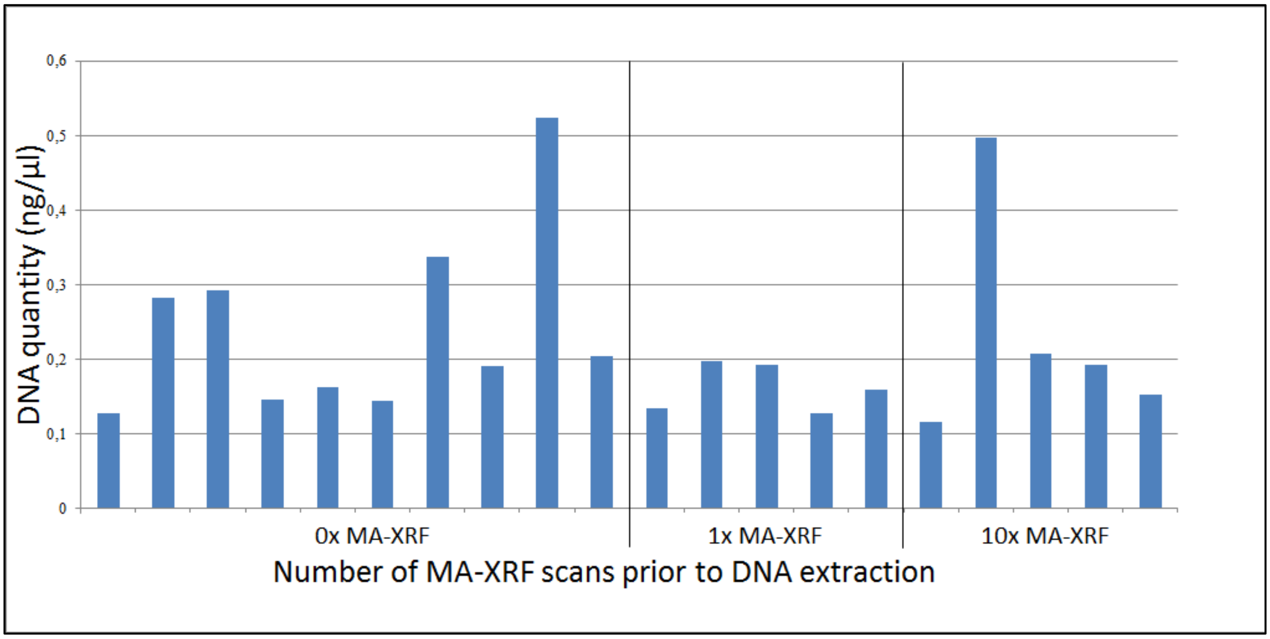

Figure S8 Spectra corresponding to the three types of ammunition.

Data retrieved from the bullet wipe area directly around the bullet holes that were created from close range (10cm or shorter) shots. (Instrument : M6, pixel scan time = 60 ms)

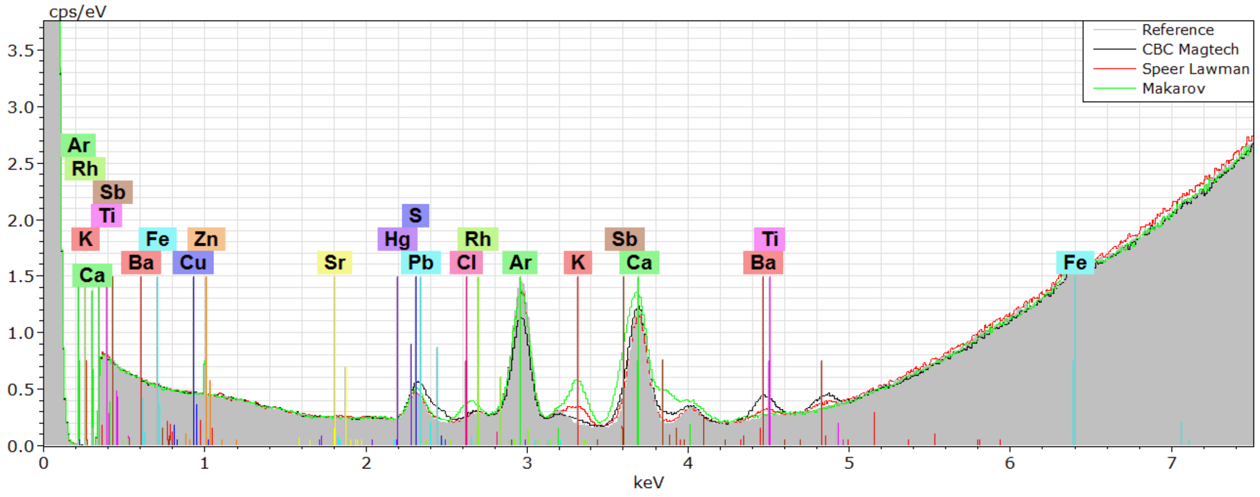

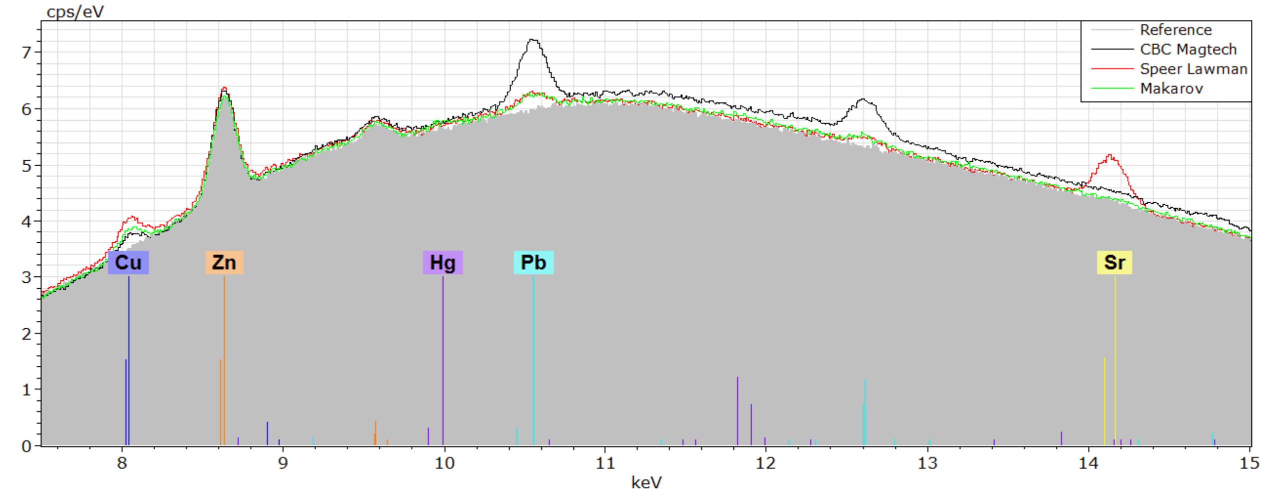

Figure S9 XRF elemental images for Cu, Pb, Ba and Sb as a function of shooting distance. The elemental images were created directly from the full MA-XRF dataset and represent an area (15 x 15 cm) encompassing the bullet holes created with CBC Magtech ammunition. Some images contain multiple patterns. The bullet hole that corresponds to the listed distance is plotted in the middle of each image. (Instrument : M6, pixel scan time = 60 ms)

	Cu	Pb	Ba	Sb
2.5cm	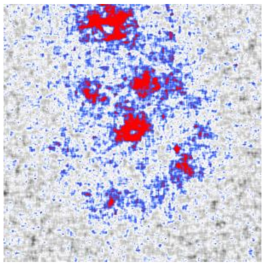	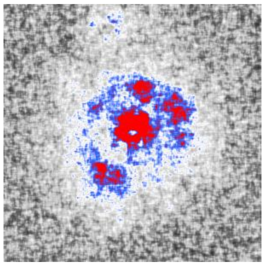	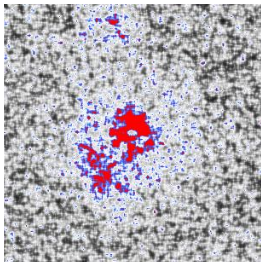	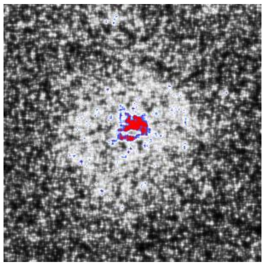
10cm	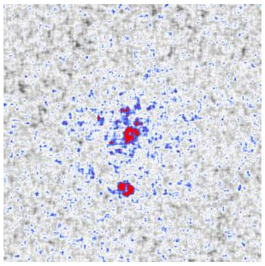	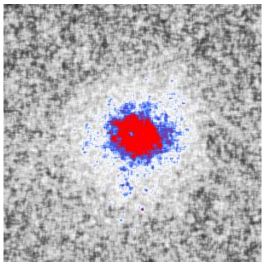	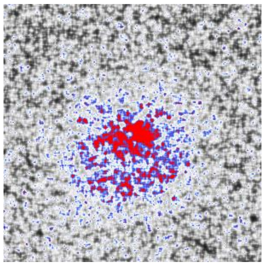	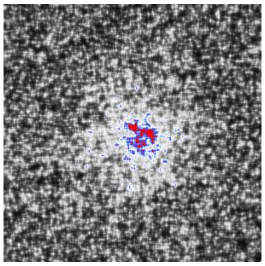
50cm	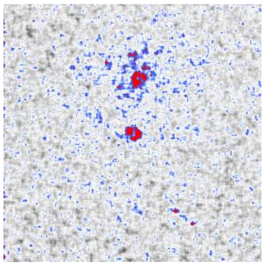	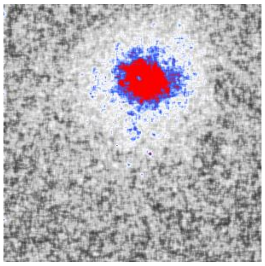	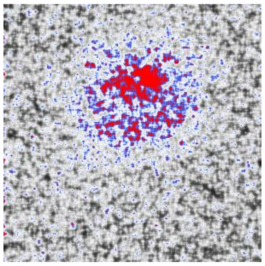	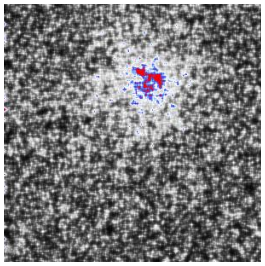
150cm	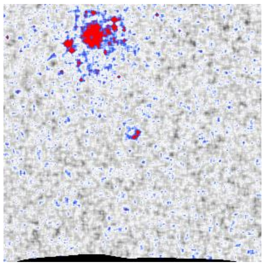	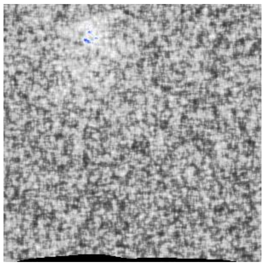	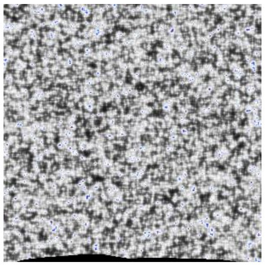	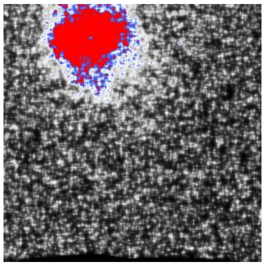

Figure S10 XRF elemental images for Sb, Cl and Cu as a function of angle of incidence. The elemental images were created directly from the full MA-XRF dataset and represent an area (10cm x 10cm) encompassing the bullet holes created with Makarov ammunition under two different angles of incidence. (Instrument : M6, pixel scan time = 60 ms)

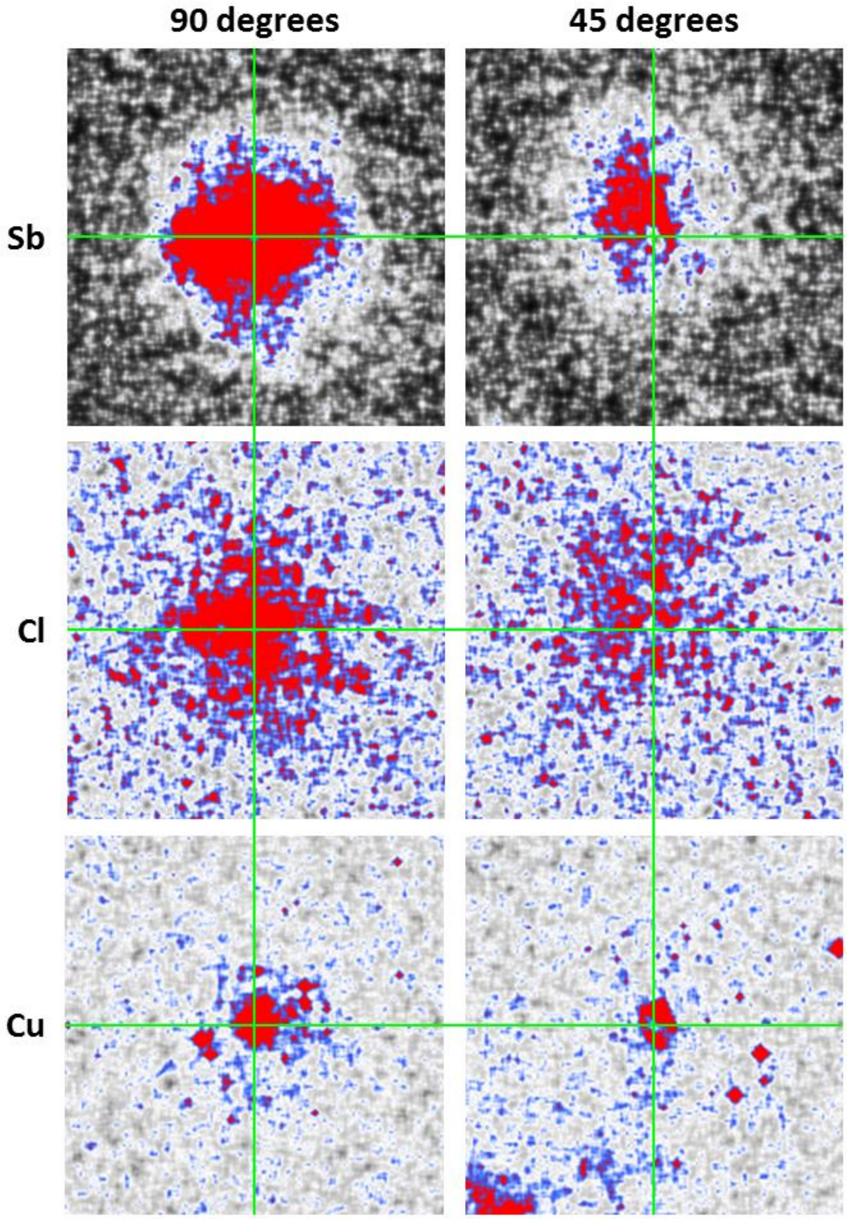
90 degrees
45 degrees
Sb
Cl
Cu

Figure S11 XRF images of Pb calibration series (for conditions see Figure S1) to illustrate the effect of overall presence and amount of elements in the scan area on the color intensity depicted in the image. Image a) contains all calibration spots and image b) depicts an area containing the 1, 10 and 100 ppm spots.

a)

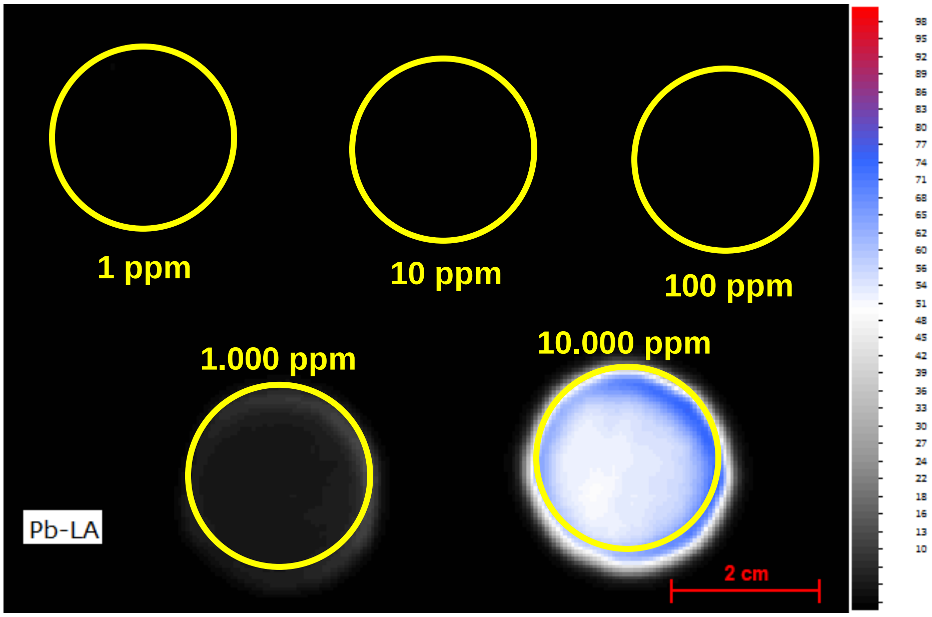

b)

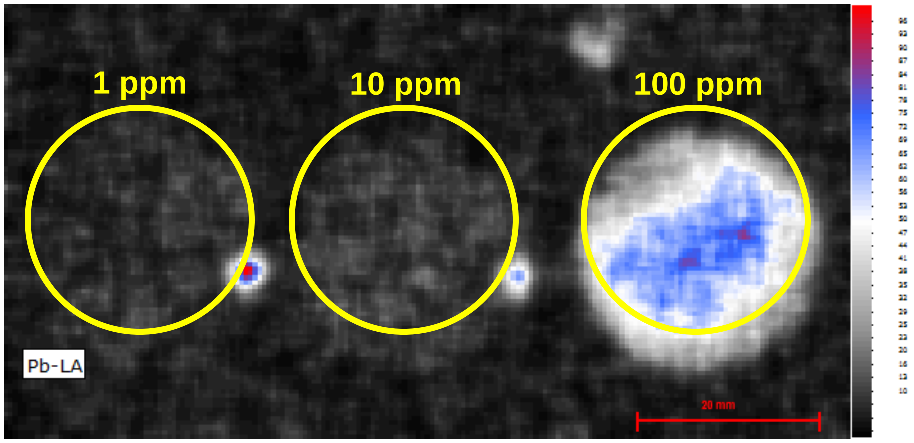

Table S3 Overview of shooting parameters during sample preparation.

ID	Firearm	Ammunition	Shooting distance and other remarks
1	Glock 17	CBC Magtech, 9mm PARA	10 cm
2	Glock 17	CBC Magtech, 9mm PARA	10 cm
3	Glock 17	CBC Magtech, 9mm PARA	50 cm
4	Glock 17	CBC Magtech, 9mm PARA	150 cm
5	Glock 17	CBC Magtech, 9mm PARA	2.5 cm
6	Glock 17	Speer Lawman Clean Fire, 9mm Parabellum	2.5 cm / memory effect CBC Magtech
7	Makarov (PM) type pistol	9mm Makarov	10 cm / 45 degrees angle of incidence
8	Makarov (PM) type pistol	9mm Makarov	10 cm

Table S4 Point measurements of Fe-Kα peak intensity in cps/eV @ 6.399 keV within a single droplet of blood (50kV, 700muA, no filter, t=100s, ambient conditions, DxSy = donor x en sample y).

Measurement	D5S1	D5S2	D5S3	D4S1	D4S2	D4S3
1	0.94	0.82	0.95	0.92	1.16	0.89
2	0.95	0.79	0.95	0.97	1.15	0.88
3	0.92	0.85	0.96	0.96	1.14	0.93
4	0.91	0.84	0.98	0.93	1.12	0.9
5	0.92	0.83	0.91	0.92	1.13	0.93
6	0.91	0.82	0.92	0.91	1.17	0.91
7	0.89	0.80	0.90	0.94	1.19	0.93
8	0.91	0.82	0.94	0.98	1.11	0.89
9	0.94	0.82	0.91	0.96	1.15	0.91
10	0.89	0.83	0.96	0.95	1.12	0.97
average	0.918	0.822	0.938	0.944	1.144	0.914
SD	0.019	0.017	0.025	0.022	0.024	0.025
RSD	**2.1**	**2.0**	**2.7**	**2.4**	**2.1**	**2.8**
min value	0.89	0.79	0.9	0.91	1.11	0.88
max value	0.95	0.85	0.98	0.98	1.19	0.93
range	0.06	0.06	0.08	0.07	0.08	0.05

References Supplementary Information

[SI.1] J. M. Harrington, D. J. Young, A. S. Essader, S. J. Sumner, and K. E. Levine, “Analysis of human serum and whole blood for mineral content by ICP-MS and ICP-OES: Development of a mineralomics method”, Biol. Trace Elem. Res., vol. 160, no. 1, pp. 132–142, 2014.

[SI.2] E. I. Hamilton, E. Sabbioni, and M. T. Van Der Venne, “Trace element reference values in tissues from inhabitants of the European Community. VII. Review of trace elements in blood, serum and urine of the Belgian population and critical evaluation of their possible use as reference values”, Sci. Total Environ., vol. 158, no. 94, pp. 165–190, 1994.

[SI.3] D. H. Owen and D. F. Katz, “A Review of the Physical and Chemical Properties of Human Semen and the Formulation of a Semen Simulant”, J. Andrology, Vol. 26, No. 4, pp. 459-469, 2005.

[SI.4] P. Apostoli, S. Porru, C. Morandi, and A. Menditto, “Multiple determination of elements in human seminal plasma and spermatozoa”, J. trace Elem. Med. Biol., vol. 11, no. 3, pp. 182–184, 1997.

[SI.5] A.H. Colagar, E.T. Marzony, M.J. Chaichi, “Zinc levels seminal plasma are associated with sperm quality in fertile and infertile men”, Nutritions Res, vol. 29, pp. 82-88, 2009.

[SI.6] K.D. Hunter and W.S. Wilson, “The effects of antidepressant drugs on salivary flow and content of sodium and potassium ions in human parotid saliva”, Archs. Oral Biolo., vol. 40, no. 11, pp. 983–989, 1995.

[SI.7] F. O. Omokhodion and J. M. Howard, “Trace elements in the sweat of acclimatized persons”, Clin. Chim. Acta, vol. 231, no. 1, pp. 23–28, 1994.

[SI.8] S.M. Shirreffs and R.J. Maughan, “Whole body sweat collection in humans: an improved method with preliminary data on electrolyte content”, The American Physiological Society, 0161-7567/97, 1997
